# Single DermaVir Immunization: Dose-Dependent Expansion of Precursor/Memory T Cells against All HIV Antigens in HIV-1 Infected Individuals

**DOI:** 10.1371/journal.pone.0035416

**Published:** 2012-05-09

**Authors:** Julianna Lisziewicz, Nyasha Bakare, Sandra A. Calarota, Dénes Bánhegyi, János Szlávik, Eszter Újhelyi, Enikő R. Tőke, Levente Molnár, Zsolt Lisziewicz, Brigitte Autran, Franco Lori

**Affiliations:** 1 Genetic Immunity, Budapest, Hungary and Genetic Immunity Inc, Mclean, Virginia, United States of America; 2 The Research Institute for Genetic and Human Therapy, Cambridge, Massachusetts, United States of America; 3 Szent László Hospital, Budapest, Hungary; 4 Laboratoire d’ Immunologie Cellulaire, Centre Hospitalier Universitaire Pitié-Salpétrière, Paris, France; McGill University AIDS Centre, Canada

## Abstract

**Background:**

The GIHU004 study was designed to evaluate the safety and immunogenicity of three doses of DermaVir immunization in HIV-infected subjects on fully suppressive combination antiretroviral therapy (cART).

**Methodology/Principal Findings:**

This first-in-human dose escalation study was conducted with three topical DermaVir doses targeted to epidermal Langerhans cells to express fifteen HIV antigens in draining lymph nodes: 0.1 mg DNA targeted to two, 0.4 mg and 0.8 mg DNA targeted to four lymph nodes. Particularly, in the medium dose cohort 0.1 mg DNA was targeted per draining lymph node via ∼8 million Langerhans cells located in 80 cm^2^ epidermis area. The 28-days study with 48-week safety follow-up evaluated HIV-specific T cell responses against Gag p17, Gag p24 and Gag p15, Tat and Rev antigens. DermaVir-associated side effects were mild, transient and not dose-dependent. Boosting of HIV-specific effector CD4^+^ and CD8^+^ T cells expressing IFN-gamma and IL-2 was detected against several antigens in every subject of the medium dose cohort. The striking result was the dose-dependent expansion of HIV-specific precursor/memory T cells with high proliferation capacity. In low, medium and high dose cohorts this HIV-specific T cell population increased by 325-, 136,202 and 50,759 counts after 4 weeks, and by 3,899, 9,878 and 18,382 counts after one year, respectively, compared to baseline.

**Conclusions/Significance:**

Single immunization with the DermaVir candidate therapeutic vaccine was safe and immunogenic in HIV-infected individuals. Based on the potent induction of Gag, Tat and Rev-specific memory T cells, especially in the medium dose cohort, we speculate that DermaVir boost T cell responses specific to all the 15 HIV antigens expressed from the single DNA. For durable immune reactivity repeated DermaVir immunization might be required since the frequency of DermaVir-boosted HIV-specific memory T cells decreased during the 48-week follow up.

**Trial Registration:**

ClinicalTrial.gov NCT00712530.

## Introduction

Currently, continuous administration of combination antiretroviral therapy (cART) is the standard care for the treatment of HIV-infected individuals. cART effectively reduces viral load and maintains undetectable HIV-RNA levels. These drugs significantly increase survival of HIV-infected people, however, patients on optimal cART still have 12 years shorter life expectancy than HIV negative people [1]. cART does not decrease viral reservoirs, including those located in gut-associated lymphoid tissues (GALT), therefore if treatment is discontinued, the virus inevitably rebounds [2], [3]. Even intensification of cART with entry-, protease- or integrase-inhibitors could neither decrease the viral reservoirs nor increase HIV-specific immunity [4], [5], [6], [7]. These results proved that despite virologic success cART alone is unlikely to cure HIV disease. It has been recently shown that the size of the viral reservoir in the GALT inversely correlated with the frequency of HIV-specific central-memory T cells [4], [8]. These data suggested that cART intensification with therapeutic vaccination aimed at expanding HIV-specific T cell pool with central-memory features bears the potential to accelerate clearance of the viral reservoir.

**Figure 1:**
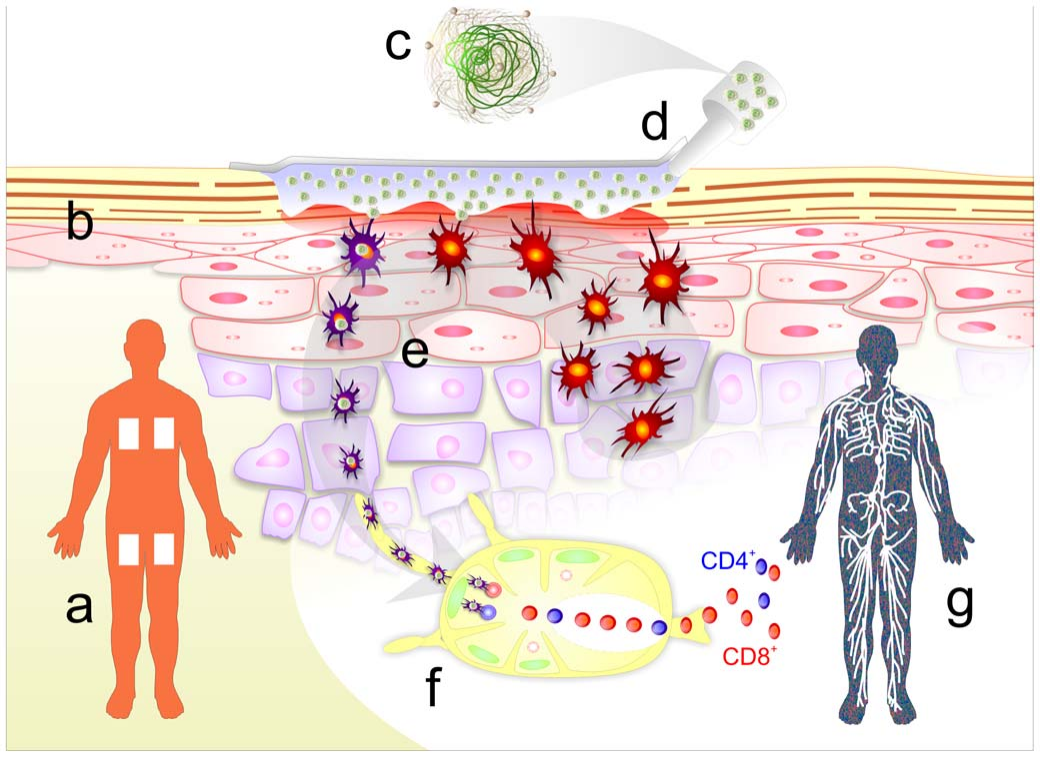
DermaVir immunization. (a) DermaVir administration area on the skin surface under 80 cm^2^ large DermaPrep patches. The immunization starts with a standardized skin preparation to interrupt the stratum corneum and activate Langerhans cells causing a mild and transient erythema. (b) Cross-sectional view of the epidermis with approximately 8 million Langerhans cells estimated under one patch. (c) DermaVir pathogen-like nanomedicine, a formulation to facilitate cellular entry, nuclear transport and expression of the plasmid DNA-encoded antigens. The nanomedicine consists of the “core” that is a condensed plasmid DNA and a mannosylated polyethylenimine “envelope” [10], [11]. (d) Needle-free, topical, Langerhans cell-targeting vaccine administration with the DermaPrep device. The special semi-occlusive patch is kept on the skin for three hours. (e) Activated epidermal Langerhans cells take up DermaVir, mature to antigen presenting dendritic cells and migrate to the draining lymph nodes. (f) In the lymph node dendritic cells express the DNA-encoded antigens: 15 HIV proteins that assemble into a complex VLP^+^ [9]. These cells present several hundreds of epitopes to naïve T cells and prime HIV-specific precursor/memory T cells. (g) HIV-specific precursor/memory T cells with high proliferative capacity circulate in the body to kill HIV-infected cells.

DermaVir is a therapeutic vaccine, different from conventional preventive vaccines aiming to protect healthy people against infections. In contrast to preventive vaccines that must induce antibody responses in uninfected subjects, therapeutic vaccines must expand the HIV-specific memory T cell pool in patients who have been already exposed to large amounts of HIV antigens and developed both antibody and T cell responses not potent enough to fully suppress viral replication. We hypothesized that it is unlikely that simply injecting additional HIV antigens would have any therapeutic effect. Therefore, we designed DermaVir as a pathogen-like synthetic nanoparticle capable to express complex Virus-like Particles (VLP^+^) in dendritic cells. These VLP^+^ antigens preserve the structure and the epitope content of the wild-type virus [9], [10], [11]. VLP^+^- expressing dendritic cells can prime naïve CD4^+^ and CD8^+^ T cells to expand the HIV-specific memory T cell pool [12], [13], [14]. Proof of concept efficacy studies performed in SIV_251_-infected macaques, some of them with AIDS, suggested that DermaVir immunization alone or in combination with cART could suppress viral load and improve survival of HIV infected people [15]. The features of DermaVir immunization are depicted in Figure 1.

Here we describe the first-in-human study conducted with the DermaVir therapeutic vaccine candidate in Budapest, Hungary. The aim of this Phase I dose escalation study was to evaluate safety and tolerability of DermaVir immunization in HIV-infected patients treated with fully suppressive cART and to compare the immunogenicity of the different DermaVir doses. T cell responses are usually measured after short-term peptide stimulation in an IFN-gamma ELISPOT assay. These T cells are thought to represent mainly effector-memory cells, which circulate shortly after antigenic priming or recall [16]. It has been previously shown that the quantity or frequency of HIV-specific T cells measured after short antigenic stimulation are not associated with better clinical outcome [17], [18], [19]. Measuring central-memory T cells, on the other hand, requires antigenic re-stimulation, therefore we utilized the PHPC (Precursors with High Proliferation Capacity) assay to measure such immune reactivity. Others and we have shown that the PHPC assay quantifies the memory T cells, a distinct population from effector T cells, and these central-memory T cells appear to play a key role in the protection from malaria, HIV and HCV [20], [21], [22], [23].

## Methods

### Study Design

The GIHU004 phase I trial was designed to evaluate the safety, tolerability and immunogenicity of DermaVir immunotherapy in individuals with chronic HIV-1 infection treated with fully suppressive cART. The protocol for this trial and supporting CONSORT checklist and flowchart are available as supporting information; see Checklist S1, Flowchart S1 and Protocol S1.

DermaVir nanomedicine was administered topically using the DermaPrep device (see below) once, at study day 0. Subjects remained in the clinic for observation for 3 hours after the immunization. After 3 hours, the DermaPrep patches were removed and the skin was evaluated. After four-week extensive safety and immunogenicity evaluations, subjects were followed up for an additional 48 weeks for safety.

Nine subjects were sequentially enrolled into each cohort. Cohort 1: three subjects received a single low-dose DermaVir immunization (0.1 mg pDNA/subject, 0.8 mL total volume of DermaVir, administered topically with DermaPrep under two skin patches). Further enrolment of subjects into the medium and high dose cohorts began only after the safety data for the low and medium doses cohorts were available and the criteria for enrolling into the next cohort were met. The main criterion to enroll the next dose cohort was the absence of a dose-limiting toxicity in any of the DermaVir subjects in the prior cohort. Cohort 2: three subjects received a medium-dose DermaVir immunization (0.4 mg pDNA/subject, 3.2 mL total, administered topically with DermaPrep under four patches). Cohort 3: three subjects received a high-dose DermaVir immunization (0.8 mg DNA/subject, 6.4 mL total, administered topically with DermaPrep under eight patches).

### Participants

The study population included nine HIV infected men and women 18 to 50 years of age with a peak plasma HIV-1 RNA >1,000 copies/mL before initiation of cART containing drugs of at least two different classes. Eligible subjects had remained on a stable cART regimen without changes or interruptions within the 24 weeks prior to study entry and had a plasma HIV-1 RNA level <50 copies/mL measured at least twice within 12 weeks prior to study entry. Subjects had a CD4^+^ cell count >300 cells/mm^3^ at the time of entry, a nadir CD4^+^ cell count >250 cells/mm^3^ and Karnofsky performance score ≥90. Exclusion criteria included infection with hepatitis viruses (HBV, HCV), adhesive sensitivity, use of immunomodulatory therapy or vaccine prior to study, treatment with topical corticosteroids at vaccination sites, excessive exposure to sun or tanning lights, and laser hair removal. Pregnant women or patients with significant laboratory abnormalities were also excluded, and all subjects were required to use an effective contraceptive method during the study. Participants were recruited from the Szent László Hospital, Budapest, Hungary.

### Ethics

The protocol and the informed consent was reviewed and approved by the Local Ethics Committee and the Hungarian National Institute of Pharmacy (OGYI) responsible for oversight of the study. All study participants signed the informed consent.

### Preparation of the Study Vaccine

The components of DermaVir were supplied by Genetic Immunity (McLean, VA) in three separate vials. Vial 1 contained 1 mg/mL pLWXu1 plasmid DNA [9]; Vial 2 contained 13.6 mM PEIm; Vial 3 contained 10% Dextrose/Glucose solution (USP) [10].

The final manufacturing step of DermaVir was performed at the clinic as described previously [10]. First the pDNA and the PEIm was diluted with the sugar solution, then the two solutions were mixed and the procedure were documented. The final DermaVir product was kept at 4°C after formulation and administered to the subject within 3 hours.

### Topical Vaccination with DermaPrep

DermaVir was administered with DermaPrep medical device (Genetic Immunity, McLean, VA). DermaPrep contains the following material in a pouch: exfoliating sponge, stripping tapes and skin patches (each for vaccination of an 80 cm^2^ area). In addition, the following materials are required for vaccine administration: disposable razors, surgical markers, alcohol swabs, syringes and needles to draw up the vaccine solutions (not for injection).

DermaPrep skin sites included upper back and ventral upper thigh. The skin site was shaved and disinfected using alcohol swab. After the skin surface dried, it was rubbed with an exfoliating sponge, then stripping tapes were applied to the skin site to remove residual cell matter from the skin surface. The skin patch was applied to the prepared skin site and the liquid vaccine was injected with a needleless syringe under the pocket formed by the patch on the surface of the skin. The pocket was then sealed and the procedure was repeated for all remaining skin sites.

Preclinical safety and reactogenicity of topical DermaVir vaccination conducted in swine and rabbits had previously demonstrated that the major side effect of DermaVir vaccination was transient mild erythema, due to the skin preparation procedure required to the enhance skin penetration. Erythema, a sign of inflammation, also provides the “danger signal” to Langerhans cells to migrate to the draining lymph nodes [24], [25]. Therefore, we do not consider transient, mild erythema during DermaVir vaccination as a toxic side effect but rather as a signal required for the efficacy of the transdermal administration process with DermaPrep.

### Study Endpoints

The primary outcome measured in GIHU004 was the occurrence of at least one grade 3 or higher adverse event including signs/symptoms, laboratory toxicities and clinical events possibly, probably or definitely related to study treatment as judged by the Principal Investigator or the site investigators during the 28 days after DermaVir administration.

The following secondary endpoints were observed: CD4^+^ and CD8^+^ T-cell counts/mL, HIV-1 RNA copies/mL <50 copies/mL, HIV-specific T cell responses; anti-DNA antibody responses, tolerability as occurrence of premature study treatment discontinuation (i.e. less than three hours of patch application at all designated sites) due to subject/physician requesting discontinuation even though no protocol-defined toxicity endpoint had been reached.

### Assessment of Safety

Safety evaluations performed at each study visit (Day 0, Day 7, Day 14, Day 28) included physical examination with local vaccine site evaluation, hematology and blood chemistry, liver function tests, urinalysis, CD4^+^ and CD8^+^ T cell counts and HIV-1 RNA. Autoantibody testing for anti-nuclear antibodies (ANA) and anti-double stranded DNA antibodies (anti-ds-DNA) was performed at baseline and at Day 28. All these assays were included in the post-treatment safety follow up evaluations performed at weeks 12, 24, 36 and 48.

### Precursor/memory T Cell (PHPC) Assay

HIV-specific T cell precursors with high proliferative capacity (PHPC) were quantified as described earlier [21]. Briefly, patients’ cryopreserved peripheral blood mononuclear cells (PBMC) were plated and stimulated with HIV-1 peptides obtained from the National Institutes of Health AIDS Research and Reference Reagent Program. Peptide pools consisted of 15 amino-acid long peptides with 11 amino-acid overlap corresponded to the complete sequence of HIV-1 Consensus B Gag (p17, p24, p15), Tat, and Rev. Positive control was PHA (Sigma-Aldrich), negative control was the culture medium stimulation. Cells were cultured for 12 days in complete culture medium supplemented with IL-2 (R & D Systems, Milan, It). On day 12, cells were tested in the same way as the ELISPOT in response to the corresponding antigen used for stimulation. PHPC assay was done in duplicates or triplicates and results are presented as PHPC counts (net spots/million PBMC).

### ELISPOT Assay

A human IFN-gamma ELISPOT kit (Diaclone, San Diego, CA) was used according to the manufacturer’s protocol. HIV-1 peptide pools (see PHPC assay) were diluted 1/200 in complete culture medium and 0.1 mL was added to 0.1 million cells into each well. PHA was the positive control and cells suspended only in complete culture medium served as a negative control. Spots were counted using automated ELISPOT reader system (A-EL-Vis). Results are presented as ELISPOT counts (net spots/million PBMC).

### ICC Assays

ICC (intra-cellular cytokine flow cytometry) assay quantifying simultaneously CD4^+^ and CD8^+^ T cells producing IFN-gamma and IL-2 was described previously [25], [26]. Peptide pools described for PHPC assay or mock were used as antigenic stimulation of 1 million PBMC for 6 h at 37°C. Brefeldin A (10 mg/mL; Sigma) was added during the last 4 h of incubation. Red blood cells were lysed and samples were permeabilized (PermFACS, Becton Dickinson, UK) and stained with FITC-, PE-, PerCP- and allophycocyanin (APC)-conjugated antibodies to IFN-gamma, IL-2, CD4 and CD8 (BD Pharmingen, UK), respectively, and fixed with 5% formaldehyde in PBS. Samples were analyzed on a FACSCalibur flow cytometer using CellQuest Pro software by gating first on the lymphocyte population identified by forward and side scatter and on CD4^+^- or CD8^+^-bright populations and then by analysis of IL-2 and IFN-gamma positive populations within the CD4^+^ and CD8^+^ lymphocyte gates. The mean frequencies of CD4^+^ or CD8^+^ T cells secreting the different cytokines were calculated from triplicate assays.

### Supersensitive HIV RNA Assay

NucliSens HIV-1 QT assay (bioMerieux, Inc. Durham NC) was used according to the manufacturer’s protocol including the improved elution of nucleic acids from silica particles and a color-controlled precipitation of eluted nucleic acids followed by isothermal transcription based nucleic acid amplification (NASBA). The lower cutoff of the NucliSens is 5 copies/mL [27].

### Statistical Analysis

Individuals in the cohorts were summarized using means and compared using two-sided Wilcoxon Rank-Sum tests. All tests were exploratory because of the small sample size.

## Results

This Phase I study enrolled nine HIV-infected adult subjects. All had durable suppression of HIV-RNA on cART over the previous 6 months and CD4 count over 300 cells/mm^3^ (Table 1). Ten subjects were screened and one subject (08) failed to meet entry criteria due to a history of bleeding and diabetes.

**Table 1:** Baseline data of study participants.

*Cohort (Code)	Gender	Age	cART	Nadir CD4	Baseline CD4	Baseline CD8	HIV RNA
1 (01)	F	50	Combivir Efavirenz	530	908	484	<5
1 (02)	M	43	Didanosine Tenofovir Kaletra	261	507	1335	<5
1 (03)	M	42	Combivir Efavirenz	350	860	789	<5
2 (04)	F	32	Combivir Efavirenz	317	626	1050	<5
2 (05)	M	39	Combivir Kaletra	296	721	823	37
2 (06)	M	39	Combivir Efavirenz	392	822	940	<5
3 (07)	M	46	Zidovudine Lamivudine Kaletra	327	1050	2047	<5
3 (09)	M	29	Combivir Efavirenz	294	524	432	<5
3 (10)	M	32	Combivir Indinavir Ritonavir	257	528	563	<5

* Cohort 1: Low dose DermaVir (0.1 mg DNA); Cohort 2: Medium dose DermaVir (0.4 mg DNA); Cohort 3: high dose DermaVir (0.8 mg DNA). All participants were Caucasian and was negative for ANA and anti-ds-DNA; F: Female; M: Male. CD4 in counts/mm^3^.

Nine subjects, in three sequential dose cohorts, received on study Day 0 a single DermaVir immunization administered topically with the DermaPrep device in three sequential cohorts. Low dose: 0.1 mg pDNA, 0.8 mL DermaVir administered under two DermaPrep patches. Medium dose: 0.4 mg pDNA, 3.2 mL DermaVir administered under four DermaPrep patches. High dose: 0.8 mg pDNA, 6.4 mL DermaVir administered under eight DermaPrep patches. Subjects were on study for a total of 28 days followed by a post-treatment safety follow-up for 48 weeks. cART was not interrupted. All subjects completed the 28-day treatment and 48 weeks safety follow up phase.

### Adverse Events

All doses of DermaVir immunization were safe and well tolerated. There was no death, no serious adverse event and no discontinuation from the study. No subject reached the primary endpoint of a grade 3 or higher adverse event including signs/symptoms, laboratory toxicities and clinical events at least possibly related to DermaVir treatment. We found that 2 of 9 subjects developed a low grade fever and 4 of 9 subjects had minor skin reactions (pruritus, scarring, skin hypersensitivity, erythema). All adverse events at least possibly related to DermaVir are shown in Table 2.

**Table 2:** All Adverse Events judged attributable to DermaVir immunization.

DermaVir Dose Cohorts	Code	Adverse Events (Grade)	
		DermaVir-related	DermaVir-unrelated
Low (1)	01	Lumbago (1)	
	02	Fever (1)Pruritus (1)Myalgia (1)	
	03	Chills and 37.6°C fever (1)Palpable lymph nodes of left foss.Femoralis (1) Right shoulder scar caused by the tape (1)	Triglycerides (2)
Medium (2)	04	None	
	05	Fatigue (1)Skin hypersensitivity (1)	Hyperbilirubinemia (2)
	06	None	
High (3)	07	Macular Erythema (1)	Hyperbilirubinemia (2)
	09	None	
	10	Nausea (1)Eosinophilia (1)	Triglycerides (2)

There were no treatment-emergent abnormal serum chemistries, hematology values and vital signs judged to be clinically significant. One of three subjects in every cohort experienced grade 1 hematological abnormalities judged not related to study treatment. Serum chemistry abnormalities were generally grade 1, except two cART-related grade 2 abnormalities (Table 2). All participants remained negative for both ANA and anti-ds-DNA during the study. There were no differences in the frequencies of adverse events among the cohorts.

Local skin reactogenicity assessed after the patches were removed (3 hours after skin preparation and DermaVir administration) was limited to transient irritation and macular erythema of mild severity at two of the eight vaccination sites in one patient. Enlarged inguinal lymph nodes were observed in one subject in the week following vaccination that resolved by the next visit (Table 2).

### HIV-RNA, CD4 and CD8 Cell Counts

In all the subjects plasma HIV RNA levels were <50 copies/mL prior to and during the study, suggesting that DermaVir immunization was not associated with measurable viral load activation or blips. In an attempt to detect even minimal viral load decreases HIV-RNA levels were evaluated with a supersensitive PCR assay. Eight of nine patients had <5 copies/mL prior (Table 1) and all the patients had <5 copies/mL 28 days following immunization (data not shown).

CD4 counts generally increased from baseline to Week 4 and Week 48 in the low and median dose cohorts and decreased in the high dose cohort (Table 3). There were no changes in any subject’s antiretroviral drug regimen during the 48 weeks of observation.

**Table 3:** CD4^+^ and CD8^+^ T cell counts.

Cohorts (Code)	CD4 counts/mm^3^			CD8 counts/mm^3^		
	Study weeks					
	0	4	48	0	4	48
1 (01)	908	1,140	998	484	510	528
1 (02)	507	361	515	1,335	883	1,287
1 (03)	860	1,179	1,187	789	1,149	1,413
2 (04)	626	909	551	1,050	1,636	866
2 (05)	721	774	820	823	831	890
2 (06)	822	852	1,043	940	1,113	911
3 (07)	1,059	834	901	2,047	1,222	1,650
3 (09)	524	528	443	432	455	488
3 (10)	528	501	633	563	509	680

### HIV-specific T Cells Detected After Short-term Antigenic Stimulation

HIV Gag, Tat and Rev specific ELISPOT responses detected during the study are shown in Table 4. We considered as responders the subjects having ≥10 fold higher ELISPOT responses compared to baseline (bold labeled). Generally, the pre-immunization (baseline) values to HIV antigens were low as expected after long-term fully suppressive cART [28]. DermaVir boosted ELISPOT responses in 2 out of 3 subjects in the low dose cohort and all 3 subjects in the medium dose cohort. All the responder had Gag-specific T cells. There were no responses to any antigens in the high dose cohort.

**Table 4:** HIV-specific ELISPOT responses.

		Cohort 1			Cohort 2			Cohort 3		
		01	02	03	04	05	06	07	09	10
**Gag p17**	(0)	0	**25**	10	**10**	20	**5**	30	35	50
(weeks)	(4)	**80**	**0**	7	**330**	80	**65**	0	50	0
	(48)	**85** *	**255** *	0*	**325**	40	**370**	35	15	15
**Gag p24**	(0)	**0**	30	14	5	**0**	**5**	45	110	95
(weeks)	(4)	**290**	5	20	5	**390**	**920**	65	270	0
	(48)	**1475** *	0*	43*	10	**895**	**1370**	0	80	40
**Gag p15**	(0)	**10**	255	**24**	**5**	5	**10**	15	20	155
(weeks)	(4)	**30**	10	**30**	**45**	25	**210**	10	65	10
	(48)	**305** *	340*	**100** *	**130**	20	**830**	0	55	70
**Tat**	(0)	**0**	30	7	**0**	**0**	**0**	20	0	200
(weeks)	(4)	**10**	0	17	**5**	**35**	**40**	0	40	15
	(48)	**15** *	55*	27*	**35**	**55**	**320**	55	0	45
**Rev**	(0)	1	35	10	**0**	**0**	5	5	0	90
(weeks)	(4)	10	0	0	**5**	**5**	0	90	5	0
	(48)	0*	15*	0*	**30**	**5**	40	10	0	5

ELISPOT SFU/10^6^ PBMC are the average of a duplicate or triplicate assay; Mocks were subtracted.

* Cohort 1 samples were collected at week 24. Bold: ELISPOT responders: ≥10 fold above the response at baseline.

We also measured antigen-specific T cell responses with the ICC assay to detect the responding CD4^+^ and CD8^+^ T cell population secreting IFN-gamma and IL-2 (Table 5). Generally, the percentages of cytokine secreting T cells within the T cell populations were low. However, we found that ELISPOT responders of the medium dose cohort were also ICC responders. These subjects had both CD4^+^ and CD8^+^ T cell responses to Gag, Tat and Rev stimulation. Some of the CD8^+^ T cells produced both IFN-gamma and IL-2, the CD4^+^ T cells mostly IL-2 cytokines. In the low dose and high dose cohorts we could only sporadically detect T cell responses. The lack of HIV-specific ICC responses in the low dose cohort despite positive ELISPOT reflects the lower sensitivity of the ICC assay.

**Table 5:** HIV-specific ICC responses.

Cohorts (Code)	Gag p17		Gag p24		Gag p15		Tat		Rev	
	CD8	CD4	CD8	CD4	CD8	CD4	CD8	CD4	CD8	CD4
1 (01)	–	–	–	–	–	–	–	–	–	–
1 (02)	–	–	–	–	–	–	–	–	–	–
1 (03)	IFN	–	–	–	–	–	–	–	–	–
2 (04)	IFN IL2	–	–	–	IFN IL2	–	IFN IL2	IL2	IFN IL2	IFN
2 (05)	IL2	IL2	IL2	IL2	IL2	IL2	IL2	IL2	IL2	IL2
2 (06)	IL2	IL2	IL2 IFN	–	IL2 IFN	IL2	IL2	IL2	IL2 IFN	IL2
3 (07)	IFN	–	–	IFN	–	–	–	–	–	–
3 (09)	IFN	–	IFN	–	–	–	–	–	–	–
3 (10)	IL2	–	–	–	–	–	–	–	IFN IL2	–

Responders: at least 2 separate visits >baseline and/or response is ≥2% (net cytokine producing CD4 or CD8 T cells).

### HIV-specific Precursor/Memory T Cells

**Figure 2:**
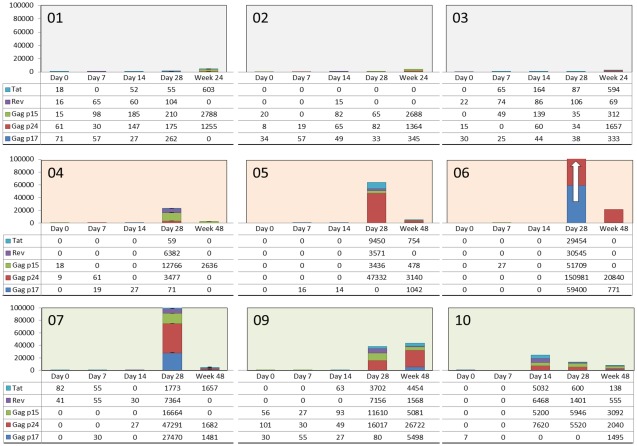
Kinetics of the HIV-specific PHPC responses. Long-lived HIV-specific precursor/memory T cells with high proliferative capacity prior and after a single DermaVir immunization in each HIV-infected individual on fully suppressive cART. 1^st^ raw: Low dose; 2^nd^ raw: Medium Dose; 3^rd^ raw: high dose DermaVir immunizations. Frequency of PHPC responses against Tat, Rev and the three Gag antigens are shown prior (Day 0) and after DermaVir immunization (day 7, day 14, day 28, and week 48). Every sample was assayed in duplicate or triplicate, and data are shown as PHPC counts/10^6^ PBMC.

HIV-specific precursors with high proliferative capacity (PHPC) were evaluated before and after single DermaVir immunization. Figure 2 demonstrates the kinetics of the PHPC responses to Gag p17, Gag p24, Gag p15, Rev and Tat of every subjects measured before immunization (week 0) and 1, 2, 4, and 48 weeks after immunization. All subjects had consistent low or undetectable PHPC counts prior to DermaVir immunization, averages being between 15 and 36 counts (net spots/million PBMC) per peptide pools. We found that DermaVir immunization induced PHPC responses in every patient within 28 days. In the low dose cohort there was a modest (+325) immune boosting (Table 6). In striking contrast, DermaVir immunization boosted the PHPC responses by 136,202- and 50,759 counts compared to baseline in the medium and in the high dose cohorts, respectively. We found statistically significant PHPC increases between baseline and day 28 for the nine subjects for total Gag, Gag p24, Gag p15, Rev, Tat (p≤.01) and Gag p17 ((p≤.05). This statistically significant increase of immune responses after vaccination of HIV-infected people demonstrates the immunogenicity of the study vaccine. The high dose cohort responses did not exceed the medium dose ones, albeit the differences between the PHPC responses of the two cohorts were not statistically significant. The PHPC counts were higher in the medium and high dose cohorts compared to the low dose at day 28 (p≤.1). The strongest responder was subject 06, who had undetectable PHPC responses to every assayed antigens prior to DermaVir immunization and developed new T cell responses: ∼30,000 counts to both Tat and Rev, ∼50,000 counts to both Gag p17 and Gag p15 and ∼150,000 counts to Gag p24. Although it generally took 4 weeks following a single DermaVir vaccination to detect new precursor/memory T cells, the responses were durable up to one year. All samples of each patient were assayed from frozen PBMC specimen in duplicates or triplicates at the same time by the same operator and with the same reagents.

**Table 6:** Increase of HIV-specific memory/precursor T cells from baseline after a single DermaVir immunization.

Dose		Antigen	Mean PHPC counts (net spots/million PBMC) (SD)**							
Low (1)			*Baseline* *		*Increase at Day 28*		*Increase at Week 24*			
	Total Gag		**85**	(55)	+226	(238)	+3,496	(1,095)		
	p17		45	(23)	+66	(108)	+181	(218)		
	p24		28	(29)	+69	(48)	+1,397	(227)		
	p15		12	(11)	+92	(90)	+1,918	(1,391)		
	Rev		**13**	(11)	+57	(50)	+10	(33)		
	Tat		**6**	(10)	+41	(44)	+393	(340)		
	**Gag+Rev+Tat** ***		**104**	(67)	**+324**	(266)	**+3,899**	(869)		
**Medium (2)**			***Baseline*** *		***Increase at Day 28***		***Increase at Week 48***			
	Total Gag		**9**	(15)	+109,715	(133,082)	+9,627		(10,429)	
	p17		**0**	(0)	+19,824	(34,274)	+604		(541)	
	p24		**3**	(5)	+67,260	(75,749)	+7,990		(11,239)	
	p15		**6**	(10)	+22,631	(25,609)	+1,032		(1,394)	
	Rev		**0**	(0)	+13,499	(14,829)	0		(0)	
	Tat		**0**	(0)	+12,988	(15,013)	+251		(435)	
	**Gag+Rev+Tat** ***		**9**	(15)	**+136,202**	(162,287)	**+9,878**		(10,257)	
**High (3)**			***Baseline*** *		***Increase at Day 28***		***Increase at Week 48***			
	Total Gag		**65**	(106)	+43,468	(42,301)	+15,632			(18,684)
	p17		**12**	(16)	+9,171	(15,847)	+2,812			(2,300)
	p24		**34**	(58)	+22,909	(21,746)	+10,114			(14,296)
	p15		**19**	(32)	+11,388	(5,361)	+2,706			(2,535)
	Rev		**14**	(24)	+5,293	(3,372)	+694			(813)
	Tat		**27**	(47)	+1,998	(1,574)	+2,056			(2,198)
	**Gag+Rev+Tat** ***		**106**	(91)	**+50,759**	(44,792)	**+18,382**			(21,477)

* Calculated from data of the 2 separate PBMC specimen obtained from every subjects at screening and at day 0, just before DermaVir immunization.

** PHPC counts were calculated as follows: (Proliferation index) x (mean number of spots/million PBMC in wells from each pool of peptides - mean number of spots/million PBMC in wells with control medium). PHPC counts in response to Gag p17 pool, Gag p24 pool and Gag p15 pool were summed to calculate total Gag responses (denoted as Gag).

*** Gag+Tat+Rev responses were calculated by adding Gag, Rev and Tat responses together; it represents ∼25% of the total immunologic potential of DermaVir [9].

### Long-term Follow Up

All subjects had a 48 weeks safety follow up. There were no significant adverse events or withdrawals, and no adverse events judged to be related to or possibly related to DermaVir immunization. No subject modified cART during the course of 48 weeks safety period.

The long-term immunogenicity of a single DermaVir immunization was observed in every patient (Figure 2). The expansion of PHPC responses in cohorts 1, 2 and 3 compared to the baseline increased by 3,899, 9,878 and 18,382 counts, respectively. Within the total assayed PHPC counts statistical significant increases were detected for total Gag, Gag p17, Gag p24, Gag p15 and Tat, but not for Rev responses (p≤.01). In the low dose cohort PHPC responses increased in 6 months compared to the previous time-points. In the medium and high dose cohorts PHPC responses decreased >10 fold in 11 months in five out of six subjects.

## Discussion

The GIHU004 study demonstrated the safety and tolerability of all three DermaVir doses in HIV-infected patients treated with fully suppressive cART. DermaVir-related side effects were mild and transient, without dose limiting toxicities. Consistent with findings from the preclinical studies, skin reactogenicity was due to the skin preparation procedure, and the mild erythema induced during the skin preparation to activate the epidermal Langerhans cells was typically no longer visible when the patches were removed 3 hours later. DermaVir immunization did not increase viral load. Decrease of HIV-RNA could be only detected in one subject because all other subjects had HIV-RNA level <5 copies/mL prior to immunization. CD4 counts increased in the low and medium dose cohorts, no dose-relationship was observed.

Long-term successful cART results in the suppression of antigen concentration and the decrease of HIV-specific T cells [27]. Accordingly, we found in our study population very low or undetectable HIV-specific T cell responses measured by ELISPOT, ICC and PHPC assays prior to DermaVir immunization. These results confirmed that optimal cART suppresses the body’s own defense mechanism that would be necessary to eliminate the infected cells remained in the reservoirs [4], [8].

The most striking result of DermaVir immunization was the dose-dependent expansion of HIV-specific precursor/memory T cells with high proliferation capacity. In the low dose cohort we have consistently found low frequency of PHPC responses suggesting that suboptimal amount of DermaVir DNA expressed in the lymph nodes. In the medium and high dose cohorts we found very high frequency of PHPC responses: for all five HIV antigen stimulation the precursor/memory T cells significantly expanded, sometimes over 2,000 fold compared to baseline. All the immunogenicity data (PHPC, ICC and ELISPOT) demonstrated the absence of additional benefit after increasing the DermaVir dose from the medium to high dose. DermaVir’s immune reactivity was slow, peaking four weeks after immunization. Such delayed kinetics is consistent with the naïve T cell priming and with the conversion to antigen-specific central memory T cells [29].

DermaVir dose escalation involved not only the administration of more vaccine. We investigated three options: (i) increase the DermaVir dose on the same skin surface area (low to medium dose), (ii) use different location of the skin to target the vaccine to different lymph nodes (low to medium dose), and (iii) increase the skin surface area to target more vaccine containing Langerhans cells to the same draining lymph nodes (medium to high dose). In the low dose we targeted 0.1 mg DNA to two lymph nodes. In medium and high doses we have targeted 0.4 mg and 0.8 mg DNA in four lymph nodes, respectively. We found that simultaneous T cell priming in different lymph nodes was required to induce the highest frequency of HIV-specific T cells. Based on the immunological outcome of the study (Tables 4, 5 and 6), we concluded that the optimal boosting of T cell responses by DermaVir was achieved in the medium dose cohort. In the medium dose cohort 0.1 mg DNA was targeted per draining lymph node via ∼8 million Langerhans cells located in 80 cm^2^ epidermis area. Doubling the amount of antigens per lymph nodes (high dose) did not increase the T cell responses. Decreasing the amount of antigens per lymph nodes (low dose) resulted in lower quantity of immune responses.

DermaVir induced precursor/memory T cell responses are particularly interesting since they have functional characteristics of central-memory T cells. In macaques DermaVir has induced potent CD4^+^ and CD8^+^ T cells with similar central-memory phenotype [14]. PHPC immune reactivity is associated with low plasma viremia and preserved CD4^+^ T cell counts and is quantitatively and qualitatively different from the T cells measured by the ELISPOT assay [21]. In this clinical study we could not phenotype DermaVir-induced T cells, however the following data suggested that they have HIV-specific central-memory T cell properties: (i) they are new HIV-specific T cells (compared to pre-immunization baseline); (ii) they are HIV-specific (mock control was subtracted); (iii) they are capable to extensive proliferation on cognate antigen encounter (12 days in the PHPC assay); (iv) they secrete IL-2 (shown by the ICC assay); (v) they appear in the peripheral blood 4 weeks after DermaVir immunization and, (vi) they persist >48 weeks after DermaVir immunization in HIV-infected people, albeit in lower quantity. These clinical findings support our hypothesis that immune responses induced by DermaVir that expresses VLP^+^ in the dendritic cells of the lymph nodes are different than vaccines targeting the somatic cells, e.g. those employing viral vectors or injected/electroporated DNA [30].

DermaVir induced precursor/memory T cells had broad specificity. We measured DermaVir’s immune reactivity with five overlapping peptide pools representing regulatory HIV proteins that are expressed very early during the viral life cycle and structural HIV proteins that are expressed late. However, the total T cell responses boosted by DermaVir were not limited to these antigens. DermaVir’s active pharmaceutical ingredient is a novel plasmid DNA (pLWXu1) that expresses all 15 HIV antigens and consequently has the highest epitope coverage within HIV vaccine candidates [9]. Gag p24 expression, also used to assess the potency of DermaVir, quantifies both the expression of all the regulatory and structural HIV proteins from the plasmid DNA and the release of a VLP^+^ [9]. By calculating the T cell responses induced by DermaVir we found that the five peptide-pools only measured approximately 25% of the total T cell responses potentially induced by DermaVir [9]. Since beside Gag p24 we also measured very potent induction of Gag p17, Gag p15, Tat and Rev-specific precursor/memory T cells, we speculate that DermaVir induced additional T cell responses specific to Protease, Reverse transcriptase, RNase, Integrase (truncated), Env gp120, Env gp41, Nef (truncated), Vif, Vpu and Vpr in the medium and in the high dose cohorts.

Initiation of therapy early in the course of HIV infection has been recently suggested to preserve immune functions and improve long-term outcomes [31], [32]. However, one limitation of early cART is the decrease of HIV-specific immunity. We propose cART intensification with DermaVir to boost the long-lived HIV-specific precursor/memory T cell pool. Our data suggest that maintenance of such immune reactivity might require repeated DermaVir immunization. This concept is supported by recent findings showing that the half-life of central-memory T cells is between 28 and 100 days in HIV infected subjects and these cells have shorter half-life and reduced frequency, particularly in those with high viral load [33]. The antiviral efficacy of DermaVir must be determined in larger trials to confirm the viral load and survival benefits found in infected macaques [15]. To address HIV variability, the plasmid DNA in the DermaVir could be adapted to provide personalized treatment possibilities by matching T cell responses with epitopes presented by the patients’ cells.

All preclinical and clinical data obtained to date imply that DermaVir therapeutic vaccine candidate might provide a novel, effective and accessible treatment option for individuals infected with HIV-1.

## Supporting Information

Checklist S1 **CONSORT checklist.**
(DOCX)

Flowchart S1 **CONSORT flowchart.**
(DOCX)

Protocol S1 **Trial protocol.**
(PDF)

## References

[1] Losina E, Schackman BR, Sadownik SN, Gebo KA, Walensky RP (2009). Racial and sex disparities in life expectancy losses among HIV-infected persons in the United States: impact of risk behavior, late nitiation, and early discontinuation of antiretroviral therapy.. Clin Infect Dis.

[2] Haase AT (2005). Perils at mucosal front lines for HIV and SIV and their hosts.. Nat Rev Immunol.

[3] Shen L, Siliciano RF (2008). Viral reservoirs, residual viremia, and the potential of highly active antiretroviral therapy to eradicate HIV infection.. J Allergy Clin Immun.

[4] Hatano H, Hayes TL, Dahl V, Sinclair E, Lee TH (2011). A randomized, controlled trial of raltegravir intensification in antiretroviral-treated, HIV-infected patients with a suboptimal CD4+ T cell response. J Infect Dis..

[5] Dinosoa JB, Kima SY, Wiegandc AM, Palmerc SE, Ganged SJ (2009). Treatment intensification does not reduce residual HIV-1 viremia in patients on highly active antiretroviral therapy.. Proc Natl Acad Sci U S A.

[6] Gandhi RT, Zheng L, Bosch RZ, Chan ES, Margolis DM (2011). The effect of Raltegravir intensification on low-level residual viremia in HIV-infected patients on antiretroviral therapy: a randomized controlled trial.. PLoS Med.

[7] McMahon D, Jones J, Wiegand A, Gange SJ, Kearney M (2010). Short-course Raltegravir intensification does not reduce persistent low-level viremia in patients with HIV-1 suppression during receipt of combination antiretroviral therapy.. Clin Infect Dis.

[8] Chapuis AG, Casper C, Kuntz S, Zhu J, Tjernlund A (2011). HIV-specific CD8+ T cells from HIV+ individuals receiving HAART can be expanded ex vivo to augment systemic and mucosal immunity in vivo.. Blood.

[9] Somogyi E, Xu J, Gudics A, Toth J, Kovács A (2011). A plasmid DNA immunogen expressing fifteen protein antigens and complex virus-like particles (VLP+) mimicking naturally occurring HIV.. Vaccine.

[10] Toke ER, Lorincz O, Somogyi E, Lisziewicz J (2010). Rational development of a stable liquid formulation for nanomedicine products.. Int J Pharm.

[11] Lorincz O, Toke ER, Somogyi E, Horkay F, Chandran PL (2011). Structure and biological activity of pathogen-like synthetic nanomedicines.. http://dx.doi.org/10.1016/j.nano.2011.07.013.

[12] Lisziewicz J, Gabrilovich DI, Varga G, Xu J, Greenberg PD (2001). Induction of potent human immunodeficiency virus type 1-specific T-cell-restricted immunity by genetically modified dendritic cells.. J Virol.

[13] Lisziewicz J, Trocio J, Whitman L, Varga G, Xu J (2005). DermaVir: a novel topical vaccine for HIV/AIDS.. J Invest Dermatol.

[14] Cristillo AD, Lisziewicz J, He L, Lori F, Galmin L (2007). HIV-1 prophylactic vaccine comprised of topical DermaVir prime and protein boost elicits cellular immune responses and controls pathogenic R5 SHIV162P3.. Virology.

[15] Lisziewicz J, Trocio J, Xu J, Whitman L, Ryder A (2005). Control of viral rebound through therapeutic immunization with DermaVir.. AIDS.

[16] Sallusto F, Geginat J, Lanzavecchia A (2004). Central memory and effector memory T cell subsets: function, generation and maintenance.. Annu Rev Immunol 22:7.

[17] Betts MR, Nason MC, West SM, De Rosa SC, Migueles SA (2006). HIV nonprogressors preferentially maintain highly functional HIV-specific CD8+T cells.. Blood.

[18] Jansen CA, De Cuyper IM, Hooibrink B, van der Bij AK, van Baarle D (2006). Prognostic value of HIV-1 Gag-specific CD4+ T-cell responses for progression to AIDS analyzed in a prospective cohort study.. Blood.

[19] Schellens IMM, Borghans JAM, Jansen CA, De Cuyper IM, Geskus RB (2008). Abundance of early functional HIV-specific CD8+ T cells does not predict AIDS-free survival time.. PLoSOne.

[20] Keating SM, Bejon P, Berthoud T, Vuola JM, Todryk S (2005). Durable human memory T cells quantifiable by Cultured Enzyme-Linked Immunospot Assays are induced by heterologous prime boost immunization and correlate with protection against malaria.. J Immunol.

[21] Calarota SA, Foli A, Maserati R, Baldanti F, Paolucci S (2008). HIV-1-specific T cell precursors with high proliferative capacity correlate with low viremia and high CD4 counts in untreated individuals.. J Immunol.

[22] Flanagan KL, Lee EAM, Gravenor MB, Reece WHH, Urban BC (2001). Unique T cell effector functions elicited by plasmodium falciparum epitopes in malaria-exposed africans tested by three T cell assays.. J Immunol.

[23] Godkin AJ, Thomas HC, Openshaw PJ (2002). Evolution of epitope-specific memory CD4 T cells after clearance of Hepatitis C virus.. J Immunol.

[24] Matzinger P (2002). The danger model: a renewed sense of self.. Science.

[25] Holzmann S, Tripp CH, Schmuth M, Janke K, Franz K (2004). A Model System Using Tape Stripping for Characterization of Langerhans Cell-Precursors In Vivo.. J Invest Dermatol 122: 1165.

[26] Ondondo BO, Yang H, Dong T, di Gleria K, Suttill A (2006). Immunisation with recombinant modified vaccinia virus Ankara expressing HIV-1 gag in HIV-1-infected subjects stimulates broad functional CD4+ T cell responses.. Eur J Immunol.

[27] Notermans DW, de Wolf F, Oudshoorn P, Cuijpers HT, Pirillo M (2000). Evaluation of a second-generation nucleic acid sequence-based amplification assay for quantification of HIV type 1 RNA and the use of ultrasensitive protocol adaptations.. AIDS Res Hum Retroviruses.

[28] Casazza JP, Betts MR, Picker LJ, Koup RA (2001). Decay kinetics of Human Immunodeficiency Virus-specific CD8+ T cells in peripheral blood after initiation of highly active antiretroviral therapy.. J Virol.

[29] Koup RA, Safrit JT, Cao Y, Andrews CA, MLeod G (1994). Temporal association of cellular immune responses with the initial control of viremia in primary human immunodeficiency virus type 1 syndrome.. J Virol.

[30] Natz E, Lisziewicz J, Thalhamer J, Weiss R, Scheiblhofer S (2011). Rational design of formulated DNA vaccine: The DermaVir approach.. Gene vaccines.

[31] Kitahata MM, Gange SJ, Abraham AG, Merriman B, Saag MS (2009). Effect of early versus deferred antiretroviral therapy for HIV on survival.. N Engl J Med.

[32] Thompson MA, Aberg JA, Cahn P, Montaner JSG, Rizzardini G (2010). Antiretroviral Treatment of Adult HIV Infection: 2010 Recommendations of the International AIDS Society–USA Panel.. JAMA.

[33] Ladell K, Hellerstein MK, Cesar D, Busch R, Boban D (2008). Central memory CD8+ T cells appear to have a shorter lifespan and reduced abundance as a function of HIV disease progression.. J Immunol.

