# Supplementary material for: Single DermaVir Immunization: Dose-Dependent Expansion of Precursor/Memory T Cells against All HIV Antigens in HIV-1 Infected Individuals
Source: PLoS One. 2012 May 9;7(5):e35416. doi: 10.1371/journal.pone.0035416 (PMC3348904; doi:10.1371/journal.pone.0035416)
Supplement: Flowchart S1 — CONSORT flowchart. (DOCX) [file pone.0035416.s002.docx]

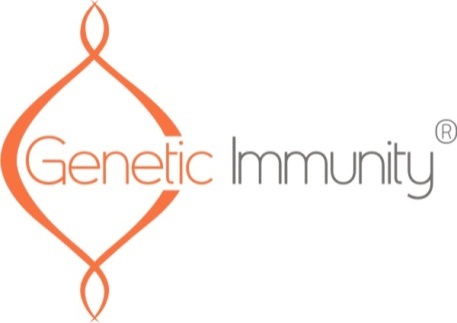


GIHU004 – CONSORT 2010 Flow Diagram

**High Dose cohort**

0.8 mg pDNA

**Medium Dose cohort**

0.4 mg pDNA

**Low Dose cohort**

0.1 mg pDNA

Analysis

Enrollment

Allocation

Follow-Up

Assessed for eligibility (n=10)

Excluded (n=1)

♦  Not meeting inclusion criteria (n=1)

♦  Declined to participate (n=0)

♦  Other reasons (n=0)

Enrolled (n=9)

Allocated to intervention (n=3)

♦  Received allocated
intervention (n=3)

♦  Did not receive allocated
intervention (n=0)

Lost to follow-up (n=0)

Discontinued intervention (n=0)

Analysed (n=3)
♦ Excluded from analysis (n=0)

Allocated to intervention (n=3)

♦  Received allocated
intervention (n=3)

♦  Did not receive allocated
intervention (n=0)

Lost to follow-up (n=0)

Discontinued intervention (n=0)

Analysed (n=3)
♦ Excluded from analysis (n=0)

Analysed (n=3)
♦ Excluded from analysis (n=0)

Lost to follow-up (n=0)

Discontinued intervention (n=0)

Allocated to intervention (n=3)

♦  Received allocated
intervention (n=3)

♦  Did not receive allocated
intervention (n=0)
