# Supplementary material for: Single DermaVir Immunization: Dose-Dependent Expansion of Precursor/Memory T Cells against All HIV Antigens in HIV-1 Infected Individuals
Source: PLoS One. 2012 May 9;7(5):e35416. doi: 10.1371/journal.pone.0035416 (PMC3348904; doi:10.1371/journal.pone.0035416)
Supplement: Protocol S1 — Trial protocol. (PDF) [file pone.0035416.s003.pdf]

GIHU004

A PHASE I STUDY TO EVALUATE THE TOLERABILITY AND SAFETY OF LC002, A DERMAVIR VACCINE, IN HIV-1-INFECTED SUBJECTS CURRENTLY UNDER TREATMENT WITH HIGHLY ACTIVE ANTIRETROVIRAL THERAPY (HAART)

Sponsored by:

Genetic Immunity, LLC

Principal Investigator:	Denes Banhegyi, M.D.
Investigator:	Janos Szlavik, M.D
CRO:	Pharmathesis Kft<br>Daniel Sztaniszlav
Final Version:<br>Amendment 1:	April 28, 2004<br>September 28, 2004

CONFIDENTIAL

PROTOCOL AUTHORIZATION

GIHU004: A PHASE I STUDY TO EVALUATE THE TOLERABILITY AND SAFETY OF LC002, A DERMAVIR VACCINE, IN HIV-1-INFECTED SUBJECTS CURRENTLY UNDER TREATMENT WITH HIGHLY ACTIVE ANTIRETROVIRAL THERAPY (HAART)

Version: September 28, 2004

[signature] 30 SEP 2004

Signature, Principal Investigator   Date

SPONSOR SIGNATURE

GIHU004: A PHASE I STUDY TO EVALUATE THE TOLERABILITY AND SAFETY OF LC002, A DERMAVIR VACCINE, IN HIV-1-INFECTED SUBJECTS CURRENTLY UNDER TREATMENT WITH HIGHLY ACTIVE ANTIRETROVIRAL THERAPY (HAART)

Version: September 28, 2004

Lisziewicz — Sept. 30, 2004

Sponsor Signatory — Date

Julianna Lisziewicz, Ph.D
President
Genetic Immunity, LLC
2233 Wisconsin Ave NW
Suite 503
Washington, DC 20007
USA

Revision chronology:

GIHU004	April 28, 2004	Original
GIHU004	September 28, 2004	Amendment 1

The main reason for this amendment is the addition of an independent, highly reputable laboratory for further evaluation of vaccine immunogenicity. This will increase the scientific value of the study, although the objectives of the study remains unchanged.

TABLE OF CONTENTS

Page

PROTOCOL AUTHORIZATION 2
SPONSOR SIGNATURE 3
SITES PARTICIPATING IN THE STUDY 6
STUDY MANAGEMENT 7
SCHEMA 9
1.0 STUDY OBJECTIVES 11
1.1 Primary Objectives 11
1.2 Secondary Objectives 11
2.0 INTRODUCTION 13
2.1 Background 13
2.2 Rationale 17
3.0 STUDY DESIGN 22
4.0 SELECTION AND ENROLLMENT OF SUBJECTS 23
4.1 Inclusion Criteria 23
4.2 Exclusion Criteria 25
4.3 Study Enrollment Procedures 26
5.0 STUDY TREATMENT 27
5.1 Regimens, Administration, and Duration 27
5.2 Product Formulation and Preparation 30
5.3 Product Supply, Distribution, and Pharmacy 33
5.4 Concomitant Medications 33
6.0 CLINICAL AND LABORATORY EVALUATIONS 35
6.1 Schedule of Events 37
6.2 Definitions for Schedule of Events 38
6.3 Special Instructions and Definitions of Evaluations 39
6.4 Off-Drug Requirements 44
7.0 TOXICITY MANAGEMENT 45
7.1 Reactions Thought Definitely, Possibly, or Probably Related to Study Vaccine 45
7.2 Antiretroviral Drugs 45
8.0 CRITERIA FOR TREATMENT DISCONTINUATION 47
9.0 STATISTICAL CONSIDERATIONS 48
9.1 General Design Issues 48
9.2 Endpoints 48
9.3 Accrual 49
10.0 DATA COLLECTION AND MONITORING AND ADVERSE EXPERIENCE

REPORTING..50
10.1 Records To Be Kept..50
10.2 Role of Data Management..50
10.3 Clinical Site Monitoring and Record Availability..50
10.4 Serious Adverse Experience (SAE) Reporting..50

11.0 HUMAN SUBJECTS..52
11.1 Institutional Local Ethical Committee and Informed Consent..52
11.2 Subject Confidentiality..52
11.3 Study Discontinuation..52

12.0 PUBLICATION OF RESEARCH FINDINGS..53

13.0 BIOHAZARD CONTAINMENT..53

14.0 REFERENCES..54

APPENDIX I: DEFINITION OF CDC CATEGORY C EVENTS..61

APPENDIX II: KARNOFSKY PERFORMANCE SCORE..63

APPENDIX III: IMMUNOLOGY: SPECIMEN COLLECTION, ASSAY DESCRIPTION..64

APPENDIX IV: TOXICITY GRADING..66

APPENDIX V: LC002-DERMAVIR FORMULATION RECORD..76

APPENDIX VI: DECLARATION OF HELSINKI..78

SITES PARTICIPATING IN THE STUDY

St. Laszlo Hospital

Budapest
Hungary

STUDY MANAGEMENT

Principal Investigator:

Denes Banhegyi, MD
Head, 5th Department of Medicine,
Saint Laszlo Hospital
Gyali ut 5-7, Budapest
Hungary, H-1097
Tel: +3614558152
Fax: +3614558254
Email: immunol@mail.datanet.hu

Investigator

Janos Szlavik, M.D.
5th Department of Medicine,
Saint Laszlo Hospital
Gyali ut 5-7, Budapest
Hungary, H-1097
Tel: +3614558152
Fax: +3614558254

Study Nurse

Barbai Kornélia
5th Department of Medicine,
Saint Laszlo Hospital
Gyali ut 5-7, Budapest
Hungary, H-1097
Tel: +3614558152
Fax: +3614558254

Sponsor Representative

Genetic Immunity, LLC
Dr. Jozsef Gaal
Baross u 53
1174 Budapest
Tel: 361 258 4078
Fax: 361 258 7416
Email: gaa13248@helka.iif.hu

CRO

Pharmathesis Kft.
Daniel Sztaniszlav

Monitor

Dr. Monika Eckschmied
Pharmathesis Kft
Karinthy Frigyes ut 4-6. II./1.A

1029 Budapest
Tel: 361 209 3918
Fax: 361 209 3917
Email: cro@pharmathesis.hu

FOR QUESTIONS CONCERNING SERIOUS ADVERSE EVENTS, CLINICAL MEDICAL MANAGEMENT, INCLUDING ENTRY CRITERIA, TOXICITY MANAGEMENT, CONCOMITANT MEDICATIONS, AND CO-ENROLLMENT, CONTACT THE PRINCIPAL INVESTIGATOR:

- **Dr. Denes Banhegyi**
- **Tel: 455-8192**

SCHEMA

A PHASE I STUDY TO EVALUATE THE TOLERABILITY AND SAFETY OF A LC002, A DERMAVIR VACCINE, IN HIV-1-INFECTED SUBJECTS CURRENTLY UNDER TREATMENT WITH HIGHLY ACTIVE ANTIRETROVIRAL THERAPY (HAART)

DESIGN:

GIHU004, a phase I sequential dose escalation cohort study is designed to evaluate the safety and immunogenicity of three dosing regimens of LC002 for the treatment of individuals with chronic HIV-1 infection who have highly active antiretroviral therapy (HAART)-induced durable suppression of viral replication. Subjects will be assigned to one of three cohorts. Subjects in cohort 1 will be randomized to receive one low-dose LC002 vaccination (3 subjects). Further enrollment of subjects into the medium dose cohort 2 (3 subjects) and high dose cohort 3 (3 subjects) will begin only after the safety data for cohort 1 and 2, respectively, are available, and the criteria for enrolling the next cohort are met. The main criterion to advance to the next cohort will be the absence of a dose-limiting toxicity.

DURATION:

Subjects will be on study for a total of 4 weeks followed by an additional 48 weeks for safety evaluations.

SAMPLE SIZE: 9 subjects (3 subjects/cohort)

POPULATION:

- HIV-infected men and women 18 to 50 years of age with a peak plasma HIV-1 RNA level > 1000 copies/mL before initiation of HAART
- On a stable HAART regimen (containing drugs of at least two different classes) without changes or interruptions for at least the 24 weeks prior to study entry
- With a plasma HIV-1 RNA level of < 50 copies/mL of plasma HIV-1 RNA at least twice within the 12 weeks prior to study entry
- With a CD4+ cell count > 300 cells/mm^3 within 12 weeks prior to study entry and a nadir CD4+ cell count > 250 cells/mm^3

<u>REGIMEN:</u>
Subjects will be sequentially enrolled into each cohort:

- Cohort 1:
  - Three subjects will receive a single low-dose vaccination (0.1 mg DNA/subject, 0.8 mL total, administered over two skin sites of 80 cm^2 each, 0.4 mL/site) at study day 0.

- Cohort 2:
  - Three subjects will receive a medium-dose vaccination (0.4 mg DNA/subject, 3.2 mL total, administered over four skin sites of 80 cm^2 each, 0.8 mL/site) at study day 0.

- Cohort 3:
  - Three subjects will receive a high-dose vaccination (0.8 mg DNA/subject, 6.4 mL total, administered over eight skin sites of 80 cm^2 each, 0.8 mL/site) at study day 0.

1.0 STUDY OBJECTIVES

1.1 Primary Objectives

1.1.1. To evaluate the safety of three different doses of LC002 in HIV-infected patients on HAART.

1.2 Secondary Objectives

1.2.1. To explore the immunogenicity of a LC002 for the treatment of individuals with chronic HIV-1 infection and HAART-induced durable suppression of viral replication.

1.2.1.1. To evaluate CD4+ and CD8+ T cell counts

1.2.1.2. To describe the distribution of HIV-specific immune responses with the following assays:

- Total HIV-specific lymphocyte count (CD3+ T-cells) using flow cytometric assay to detect antigen-specific IFN-gamma-producing cells that respond to whole Zn-finger-inactivated virus stimulation.
- HIV-specific CD8+ T cell count (CD8+, CD3+) using flow cytometric assay to detect antigen-specific IFN-gamma-producing cells that respond to whole Zn-finger-inactivated virus stimulation (1).

1.2.1.3. To explore whether LC002 treatment augments the magnitude of HIV-1 specific T-cell responses.

1.2.1.4 To explore whether increasing the dose of LC002 has greater efficacy in augmenting HIV specific T-cell responses in HIV-1 infected subjects.

1.2.1.5 To explore whether there are differences in longitudinal profiles of HIV-specific T-cell levels (and changes in T-cell levels from baseline) between treatment arms.

1.2.2. To monitor anti-DNA antibody response after immunization

1.2.3. To explore whether there are differences in the tolerability of LC002 with respect to pre-mature treatment discontinuation between treatment arms.
1.2.4. To explore if immunization results in any changes in viral load.

2.0 INTRODUCTION

2.1 Background

The advent of highly active antiretroviral therapy (HAART) has drastically changed the treatment of subjects with HIV infection, enabling control of viral replication over periods ranging from months to years (2). HAART-induced inhibition of HIV viral replication, in turn, is associated with a dramatic reduction in morbidity and mortality due to opportunistic infections and other complications of advanced HIV infection (3). However, cost, long-term toxicity, and the inability of many subjects to comply with an often complex regimen of medications for prolonged periods are all elements that make the current forms of HAART less than ideal as the sole modality of treatment for HIV infection. Furthermore, treatment with current HAART regimens cannot eradicate HIV from viral reservoirs within a clinically useful time frame (4, 5), requiring subjects to commit to a pharmacological treatment of indefinite duration.

Role of the immune system in HIV disease and restoration of HIV-specific immune responses as a therapeutic strategy

Lymphocyte proliferative responses (LPRs) to HIV antigens are either absent or of small magnitude in HIV-infected subjects, even at early stages of HIV infection (6), when vigorous proliferative responses to recall antigens are still seen (7). While these responses diminish in most persons with early HIV-1 infection (8), persistence of these responses appears to correlate with control of viral replication in untreated subjects (9) and, therefore by inference, with prognosis. The precise mechanism for this decreased HIV-specific helper cell response is unknown, but apoptosis (10), anergy, and deletion of HIV-reactive CD4+ clones at the site of antigen presentation, and HIV-1 replication have been proposed (11, 12). CD8+ T cells, in particular, seem to have an impaired ability to mature adequately in HIV infection (13). A number of observations in both primate models and human subjects further support the notion that SIV/HIV-specific CD8+ T cell responses play an important role in the control of viral replication and progression of disease:

- At least some subsets of “long-term nonprogressors” (14-17) and of “high-risk seronegatives” (18) maintain HIV-specific T-cell mediated immune responses, suggesting that a vigorous immune response against HIV can partially protect against disease progression in the former group and against infection in the latter. Moreover, seroconversion has been reported among some high-risk seronegatives after they have gone through periods of reduced exposure to HIV, during which they have lost markers of HIV-specific immunity (19).

- Among subjects with acute HIV infection, a delay in mounting an initial HIV-specific cytotoxic T lymphocyte (CTL) response is associated with prolonged symptoms, persistently elevated viremia, and lower CD4+ T-cell counts (20).

- Macaques immunized with a DNA vaccine expressing SIVmac239 *gag* and HIV-1 89.6 *env* augmented by IL-2 and Ig developed potent secondary SHIV-89.6P CTL responses and, although they became infected after a SHIV-89.6P challenge, they were able to maintain stable CD4+ T cell counts, low to undetectable set point plasma SHIV levels, and to remain free of disease for at least 140 days after challenge (21). With one exception, all of the immunized animals (n = 8) remained asymptomatic and kept control of viral replication for over 2 years after the initial challenge. Investigations of the development of viral sequence mutations revealed that just before rebound of viral replication, the virus from the single animal that subsequently experienced a breakthrough of viral replication, had developed a nucleotide mutation within the immunodominant gag p11C CTL epitope. This quasispecies rapidly replaced the wild-type population, and allowed the virus to escape immune control by CTL, resulting in rapid CD4+ T-cell depletion and eventual death from an AIDS-like illness (22).

- Depletion of CD8+ T cells in macaques by administration of the CD8-specific chimeric monoclonal antibody cM-T807 resulted in a significantly higher plasma SIV setpoint after acute SIV infection, and this correlated with substantial acceleration of the course of SIV disease. Similarly, in chronically infected macaques, depletion of CD8+ cells resulted in a rapid and marked burst in plasma viremia, which returned to baseline at the time of reappearance of SIV-specific CD8+ T cells (23). Similar results were obtained by using OKT8F as the CD8+ T-cell-depleting monoclonal antibody (24).

LC002 - DermaVir

A novel candidate DNA vaccine for topical administration, LC002, has recently been developed and is expected to induce T-cell immunity in HIV-infected individuals. LC002 is based on a novel immunogenic plasmid DNA construct that is formulated to mimic a pathogen that enters into the body via skin injury and induces immune responses. The DNA (pLWXu1) is formulated with polyethylenimine-mannose (PEIm) as the gene delivery system in a dextrose solution, that, when combined with a non-invasive skin preparation, makes it possible to transfer the DNA to epidermal Langerhans cells (LC). These pick up the vaccine and carry the DNA to the regional lymph nodes to present the DNA-encoded antigens to naïve T-cells, thus initiating HIV-specific cellular immune responses (25).

- Plasmid DNA pLWXu1

The active biologic is a plasmid DNA (26) expressing most of the viral proteins of HIV-1: gag, protease, reverse transcriptase, env, tat, rev, vif, vpr, truncated nef in order to induce T cell-mediated immune responses of broad specificity (Figure 1).

pLWXu1 is derived from a wild-type isolate, LW. The LW molecular clone is derived from a primary B clade virus known to be able to express all viral genes and replicate in T cells, macrophages, and dendritic cells (32).

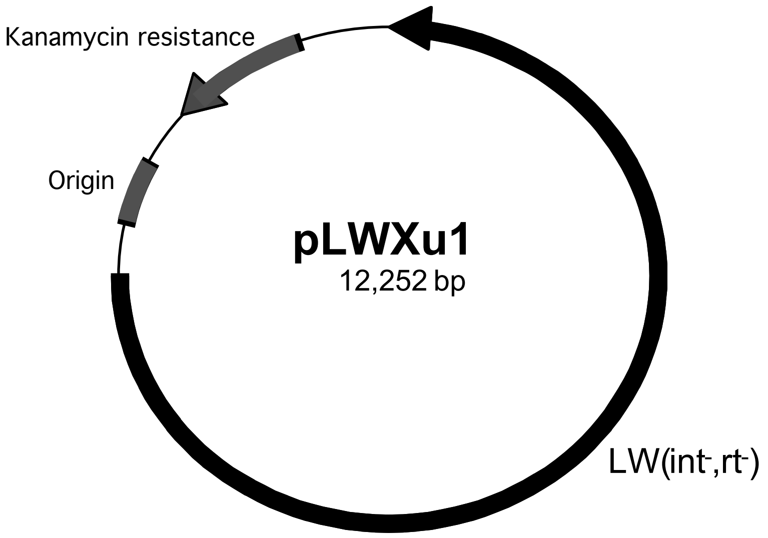

Figure: 1. *Structural Elements of pLWXu1.*

pLWXu1 consists of an HIV-1 expression cassette and structural elements required for plasmid propagation in E. coli: the origin of replication and a gene conferring resistance to kanamycin. To achieve efficient and authentic antigen presentation, HIV gene expression is regulated by the HIV-LTR.

Inactivation of reverse transcription (rt-). pLWXu1 contains a major deletion (of 633 basepairs) in the 3' LTR region, which completely impairs reverse transcription, thereby preventing DNA synthesis. Importantly, this mutation ensures a higher level of safety than mutating the gene encoding the viral reverse transcriptase (RT) because this defect cannot be rescued in trans by the patient's own HIV.

Integrase mutation (int-). To further ensure the best possible safety of the plasmid DNA, the integrase, an essential gene, has also been extensively mutated. This modification of integrase impairs its expression and disables its function (33). Indeed, in the absence of functional integrase, retroviruses cannot integrate and

replicate. Inhibition of the integrase function by a new class of antiretroviral drugs is based upon this finding.

These modifications eliminate the risk that a replication-competent virus will emerge from the plasmid upon recombination with endogenous retroviruses. Finally, the plasmid DNA does not contain any artificial sequences that could theoretically increase the pathogenicity of HIV by recombination.

DNA vaccines are a recent innovation in vaccine design because they can be used to express selected immunogenic proteins in host cells. The safety and efficacy of different DNA vaccines are currently being tested in human subjects for HIV infection and other indications. They have generally been administered by intramuscular injection or gene gun (35).

- Polyethylenimine-mannose (PEIm)

DermaVir has a particle size of ca. 100 nm diameter (mimicking a pathogen), with the DNA in complex with PEIm in dextrose solution. PEIm is essential for forming the particle as well as facilitating the transfer of plasmid DNA to LCs and is highly efficient for delivering DNA into cells in vitro and in vivo (36) (Figure 2).

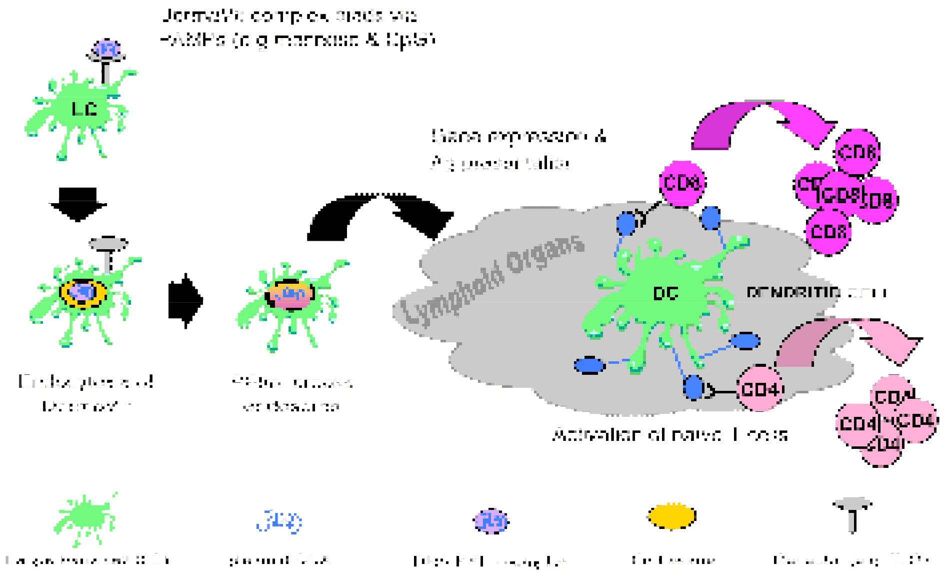

Figure 2*: Function of polyethylenimine-mannose (PEIm). PEIm is a cationic polymer capable of complexing with the anioinic plasmid DNA to form a mannosilated particle that could enter the LC via recognition of pathogen-associated molecular patterns (PAMPs), similar to a pathogen. After binding to the receptor (e.g., Toll-like receptor, TLR) on the LC, DermaVir is endocytosed. PEIm is able to break the endosome and transfer the plasmid DNA to the nucleus where transcription occurs in the LC that has matured to a dendritic cell (DC) (36, 37).*

PEI, the parental compound of PEIm, has been commercially available for more than 50 years and has been used in a broad variety of industrial applications, but it has only recently been used for the purpose of cell transfection. It is generally assumed that PEI is nontoxic. Reported oral LD50s for PEI in the mouse, rat and guinea pig range from 940 to 3300 mg/kg, and PEI caused mild irritation when an ocular dose of 500 mg was applied over 24h in rabbits (MSDS, PolyPlus-transfection). This clinical trial will test LC002 product containing lower concentrations of PEIm of s0.0006 mg per dose. A physiologically aqueous formulation of PEIm with DNA generated no toxicity after topical application in preclinical swine or macaque studies, and is not expected to cause significant toxicity in humans.

- Dextrose

The DermaVir formulation has been optimized by using 10% aqueous dextrose as a diluent. The dextrose solution is important for maintaining the size of the DNA/PEIm complex. Dextrose is not expected to cause any skin or systemic toxicity.

All the clinical materials have been formulated and packaged under aseptic conditions and tested for potency, purity, and stability.

2.2 Rationale

Hypothesis

Vaccination with DermaVir is safe and a dosing regimen for future clinical trials will be selected.

Treatment with HAART induces a significant rise in CD4+ cell counts that continues, at a slower rate, even several years after the initiation of a fully suppressive, continuous regimen (38). Although partial, the magnitude of HAART-induced immune restoration is sufficient to allow the safe discontinuation of antimicrobial prophylaxis against common opportunistic infections (39, 40). In contrast, HIV-specific CTL immune responses do not seem to be effectively restored by continuous HAART outside the acute infection period (14,41), and in fact tend to decline with time among subjects on HAART (42-44). For these reasons, the design of interventions aimed at restoring HIV-specific cellular immune responses are an obviously desirable addition to the current therapeutic armamentarium for the treatment of HIV infection. Several approaches have been investigated with this goal, most of which have attempted to enhance the host immune response via immunization with HIV antigens (45-50). Although some of these agents have been shown to stimulate immunogen-specific immunity, as evidenced by enhanced lymphoproliferative responses (LPRs) primarily

in subjects with high CD4+ T-cell counts, there has been no evidence of beneficial effect in terms of disease progression or alteration in plasma viral load (45-48, 51, 52). Clinical trials of various gp120 vaccines both in subjects with early HIV infection (ACTG 214) and subjects with later stage HIV infection (ACTG 209) have failed to show any effect on disease progression as measured by HIV-1 viral load or rate of decline of CD4+ cells, despite demonstration of immunologic response to vaccination (53). Similarly, a large multicenter trial (54) with the gpl20 depleted inactivated Remune® product failed to show a clinical benefit of the vaccine, but humoral and cell-mediated HIV-specific immunity was demonstrated in smaller cohorts of subjects.

Structured treatment interruptions (STIs), a strategy that calls for discontinuation of HAART for defined periods in order to re-expose the immune system to HIV antigens after prolonged suppression of viral replication, has been proposed as an alternative to induce the restoration of HIV-specific responses. "The Berlin patient," an individual with HIV infection who was treated with HAART before full Western blot seroconversion, was able to maintain full suppression of plasma HIV-1 RNA for at least 551 days after a voluntary second treatment interruption, in the setting of vigorous and incremental HIV-specific helper T responses, as well as a consistent CD8+ CTL response to a p17 gag epitope (55). In a group of eight subjects who started HAART after acute HIV seroconversion and achieved durable suppression of viral replication, a series of STIs resulted in enhanced CD4+ and CD8+ mediated immune responses and variable degrees of control of viral replication after prolonged periods off therapy (56). However, findings in larger cohorts of individuals treated during chronic HIV infection and undergoing STIs have shown that this strategy is rarely sufficient to attain the goal of viral suppression without antiretroviral treatment in this patient population (57).

<u>Therapeutic immunization with a dendritic cell-based topical DermaVir vaccine</u>

Dendritic cells (DCs) and their epidermal precursors, Langerhans cells (LCs), have been found to have a unique role as primers of antigen-specific immune responses (58, 59). Antigens entering the body through epithelial surfaces are captured by immature LCs and endocytosed into vesicles. If the antigen is a microbial protein, or in the presence of an adjuvant, a local innate response is induced, which leads to loss of the adhesiveness of LCs to the epidermis and migration of the antigen-loaded LCs to the draining lymph nodes via the lymphatic vessels in the dermis. In the process, LCs undergo a maturation process that involves increased expression of class II MHC and costimulatory molecules, effectively preparing the mature DCs for antigen presentation (58). In the lymph node, antigen-specific CTL activity is induced through priming of naïve CD4+ and CD8+ lymphocytes by the mature DCs, generating both effector and memory T cells, which are effective in protection against viral infection (60-65). This unique pathway lends itself to novel immunotherapeutic approaches for the treatment of HIV infection. New immune responses could be

mounted in subjects already on HAART by using DCs to present viral antigens to naïve T cells in the lymph nodes, ultimately inducing CTLs capable of killing HIV-infected cells.
Several technologies already exist to deliver antigen-presenting DCs to the lymph nodes. These include the ex vivo isolation of autologous DCs with exposure of these cells to an antigen followed by re-infusion into the animal or human host (66), as well as other methods involving delivery of naked DNA by injection or gene gun (67, 68). However, ex vivo methods are cumbersome and require highly specialized laboratories, while injection of naked DNA has resulted in only small numbers of genetically modified DC.

Initial studies using PEI/DNA complexes for transduction of cultured DCs in vitro demonstrated that genetically modified dendritic cells (GMDC) are able to induce potent HIV-specific CTLs (26). In these studies, plasmid DNA encoding replication- and integration-defective HIV-1 was introduced into monocyte-derived DCs using PEI or PEIm as the gene delivery system. After transduction, GMDC presented viral epitopes efficiently to naïve T cells, secreted IL-12, and primed both CD4+ and CD8+ HIV-specific T cells capable of producing INF-gamma and exerting potent HIV-1-specific cytotoxicity. Autologous macaque GMDC re-injected into two rhesus macaques migrated to the draining lymph node and induced CTL (26).

The immunogenicity of $DermaVir_{SHIV}$, a version of the candidate vaccine that contains a plasmid derived from pathogenic SHIV, has been demonstrated in previously uninfected non-human primates, in which a potent SIV-specific CD8+ T cell response was induced after topical administration. In this experiment, four naïve macaques were immunized with DermaVir administered to an approximately 40 cm^2 surface of the skin on four locations: the left and right upper inner thigh and left and right brachial area. The dose per location was 0.2 mL of $DermaVir_{SHIV}$, equivalent to 0.025 mg of DNA, and was maintained for 40 minutes. SIV-specific T-cell responses were absent from all the animals at the beginning of the experiment, but were easily detected in all of them, with an average of 3800 SIV-specific CD8+ cells per 10^6 CD8+ cells, 3 weeks after $DermaVir_{SHIV}$ immunization. In another study (69), two doses of $DermaVir_{SHIV}$ were administered to three macaques with late-stage SIV infection during each on-treatment period of the last 4 of 10 cycles of structured treatment interruption (3 weeks on/3 weeks off). During the first therapy interruption following the $DermaVir_{SHIV}$ treatment, the median viral rebound was 12,000 copies/mL, over 2 log less than the magnitude of the rebound observed after the previous interruption (4,292,260 copies/mL). During the following interruption the magnitude of viral load rebound further decreased from 12,000 to 460 copies/mL. Finally, during the last treatment interruption, the median viral load remained under the limit of detection of the assay (<200 copies/mL). Large quantities of SIV-specific CD8+ T cells were induced by $DermaVir_{SHIV}$ immunization in these animals, and the values were similar to those observed in animals undergoing STI-HAART early after infection (70). These

results suggested that $DermaVir_{SHIV}$ therapy induced T-cell-mediated immune responses in animals at a late stage of disease, and that these immune responses contributed to the control of viral load after treatment interruptions.

To further evaluate the vaccine in the setting of a controlled, randomized trial, 26 rhesus macaques chronically infected with SIV251 were randomized to receive 1) no therapy (control), 2) $DermaVir_{SHIV}$, 3) STI-HAART (3 weeks on/3 weeks off therapy), or 4) $DermaVir_{SHIV}$ + STI-HAART. Unlike the seven animals receiving STI-HAART alone, the seven primates randomized to receive vaccine + STI-HAART progressively controlled viral rebound during treatment interruptions from a median 33,860 copies/mL to <200 copies/mL. The six macaques treated with vaccine alone did not experience an increase in viral load, in contrast to the untreated control animals. All treated cohorts, including the vaccine alone group survived longer than the untreated controls (71).

The studies in SIV-infected monkeys described here reflect the intended dose and schedule proposed in this protocol. In infected monkeys, 15 of 16 animals received at least 7 repeated vaccinations (one more than intended for the highest dose cohort of this protocol) of 0.1 mg DNA on a skin area of 40 sq cm, with two vaccinations spaced one week apart every six weeks. One animal received only four vaccinations, and was lost due to an SIV-related B cell lymphoma, not attributable to vaccination. All vaccinated animals showed improved survival compared to the untreated controls. In addition, it should be noted that these monkeys, weighing between 4 and 10 kg, were vaccinated on a large surface area (40 sq cm) relative to their body size, but showed only mild and transient local reactions at the treated skin sites. We conclude that repeat dosing with DermaVir was not associated with significant toxicity in these monkeys.

To confirm these findings, a GLP study was performed in swine to determine the local and systemic toxicity of DermaVir. Eighteen animals were randomized into 3 groups to receive 1) dextrose solution only, 2) dextrose+PEIm only, or 3) dextrose+PEIm+DNA (DermaVir). The dose and schedule reflect one vaccination series of the highest dose cohort in this protocol: 0.4 mg DNA applied at 4 sites of 160 sq cm each, with two doses spaced one week apart. All animals were followed to four weeks after the second vaccination. There were no observations of local or systemic toxicity associated with DermaVir treatment in this study. The only side effects observed were related to the shaving and skin preparation procedure, and were mild and transient in all cases.

Given these preclinical findings, we propose to move the candidate topical DNA vaccine DermaVir into a phase I clinical trial, to evaluate its safety in humans and obtain preliminary immunogenicity data to guide further development of this product.

Rationale for dosing regimen

In the monkeys, $DermaVir_{SHIV}$, containing 0.025 mg DNA, was applied to an area of about 40 cm^2 of skin, which could contain about 2 x 10^6 LC (same numbers in mice, monkeys and humans). These LC are all potential targets for gene transfer. Since we found about 20,000 DNA expressing cells in the lymph nodes of monkeys, we estimated that at least one out of 100 LC expressed the DNA in the lymph nodes. A similar calculation in mice indicated about 1% in vivo transduction efficacy. These data confirmed our hypothesis that our formulation of DermaVir delivers DNA to lymph node dendritic cells via transduction of Langerhans cells. As a result, we determine the dose of DermaVir based on the skin area to be vaccinated.

3.0 STUDY DESIGN

This phase I trial is designed to evaluate the safety and immunogenicity of LC002 for the treatment of individuals with chronic HIV-1 infection and HAART-induced durable suppression of viral replication. The study population will include HIV-infected men and women 18 to 50 years of age with a peak plasma HIV-1 RNA > 1000 copies/mL before initiation of HAART. Eligible subjects must have been and remain on a stable HAART regimen (containing drugs of at least two different classes) without changes or interruptions within the 24 weeks prior to study entry and must have a plasma HIV-1 RNA level < 50 copies/mL at least twice within 12 weeks prior to study entry. Subjects should have a CD4+ cell count > 300 cells/mm^3 at the time of entry and a nadir CD4+ cell count > 250 cells/mm^3.

Subjects in cohort 1 will be enrolled to receive one low-dose DermaVir vaccinations (3 subjects). Further enrollment of subjects into the medium and high dose cohorts 2 and 3 (3 subjects, respectively) will begin only after the safety data for cohorts 1 and 2, respectively, are available, and the criteria for enrolling into the next cohort are met (see section 9.1.3). The main criterion to start enrolling the next dose cohort will be the absence of a dose-limiting toxicity in any of the DermaVir subjects in the prior cohort. The actual immunization will be administered once, and after the four-week treatment phase and evaluations, subjects will be followed for an additional 48 weeks for safety.

Subjects will be sequentially enrolled into each cohort:

- Cohort 1:
  - Three subjects will receive a single low-dose vaccination (0.1 mg DNA/subject, 0.8 mL total, administered over two skin sites of 80 cm^2 each, 0.4 mL/site) at study day 0.

- Cohort 2:
  - Three subjects will receive a medium-dose vaccination (0.4 mg DNA/subject, 3.2 mL total, administered over four skin sites of 80 cm^2 each, 0.8 mL/site) at study day 0

- Cohort 3:
  - Three subjects will receive a high-dose vaccination (0.8 mg DNA/subject, 6.4 mL total, administered over eight skin sites of 80 cm^2 each, 0.8 mL/site) at study day 0.

4.0 SELECTION AND ENROLLMENT OF SUBJECTS

4.1 Inclusion Criteria

4.1.1 Ability and willingness of subject or legal guardian/representative to give written informed consent.

4.1.2 HIV-1 infection, as documented by any licensed ELISA test kit and confirmed by Western blot, HIV-1 culture, HIV-1 antigen, plasma HIV-1 RNA, or a second antibody test by a method other than ELISA is acceptable as an alternative confirmatory test at any time prior to study entry.

4.1.3 On a stable antiretroviral regimen (containing drugs of at least two different classes) without changes or interruptions for at least 24 weeks prior to study entry.

4.1.4 Plasma HIV-1 RNA level of less than 50 copies/mL, while on a stable antiretroviral regimen, obtained at least twice within the 12 weeks prior to study entry.

4.1.5 Peak plasma HIV-1 RNA level before initiation of HAART > 1000 copies/mL.

4.1.6 CD4 cell count > 300 cells/mm3 within the 12 weeks prior to study entry.

4.1.7 Nadir (lowest) CD4+ cell count > 250 cells/mm3 at any time prior to study entry.

4.1.8 The following laboratory values, obtained within 30 days prior to study entry:

- Absolute neutrophil count (ANC) ≥ 1000/mm^3
- Hemoglobin ≥ 9.0 g/dL
- Platelet count ≥ 50,000/mm^3
- Serum creatinine ≤ upper limit of the laboratory normal range (ULN)
- AST (SGOT), ALT (SGPT), and alkaline phosphatase ≤ 2.5 x ULN
- Total bilirubin ≤ 2.5 x ULN

- Anti-nuclear antibody (ANA) titer of 1:40 or lower and negative for serum anti-double-stranded DNA antibody (anti-ds-DNA) test result at screening.

4.1.9 All women of reproductive potential (who have not reached menopause or undergone hysterectomy, oophorectomy, or tubal ligation) must have a negative urine β-HCG pregnancy test performed within 14 days prior to study entry.

Female study volunteers who are not of reproductive potential (who have reached menopause or undergone hysterectomy, oophorectomy, or tubal ligation) or whose male partner has undergone successful vasectomy with documented azoospermia or has documented azoospermia for any other reason, are eligible without requiring the use of contraception. Acceptable documentation of menopause, sterilization, and azoospermia is written or oral documentation communicated by clinician or clinician's staff of one of the following:

- Physician report/letter
- Operative report or other source documentation in the patient record
- Discharge summary
- Laboratory report of azoospermia (required for acceptable documentation of successful vasectomy)
- FSH measurement elevated into the menopausal range as established by the reporting laboratory.

4.1.10 All subjects must not participate in a conception process (e.g. active attempt to become pregnant or to impregnate, sperm donation, in vitro fertilization) and, if participating in sexual activity that could lead to pregnancy, the study volunteer/ partner must use two reliable methods of contraception simultaneously while receiving the protocol-specified vaccination(s) and for 3 months after the last vaccination.

A Combination of TWO of the following methods must be used:

- Condoms[1] (male or female) with or without a spermicidal agent.
- Diaphragm or cervical cap with spermicide
- IUD[2]
- Hormonal-based contraception

[1] Condoms are recommended because their appropriate use is the only contraception method effective for preventing HIV transmission.

[2] An IUD is an adequate method of birth control, but increases the risk of pelvic inflammatory disease.

4.1.11 Karnofsky performance score (Appendix II) ≥ 90 within 30 days prior to study entry.

4.1.12 Men and women age 18-50 years.

4.2 Exclusion Criteria

4.2.1 Viral load measurement > 50 copies/mL within the last 12 weeks prior to study entry.

4.2.2 History of or evidence of active skin disease (atopic dermatitis, psoriasis, etc.), chronic autoimmune disease or any other significant active skin disease.

4.2.3 Treatment with topical corticosteroids in close proximity to the proposed vaccination sites within 2 weeks prior to study entry.

4.2.4 Excessive exposure to the sun (e.g., sunbathing, tanning bed) within 2 weeks prior to study entry.

4.2.5 Use of any local skin treatments to the targeted vaccination sites within 7 days prior to study entry.

4.2.6 History of diabetes and bleeding disorders.

4.2.7 Previous CDC category C event as defined in Appendix I.

4.2.8 Pregnancy or breast-feeding.

4.2.9 Use of immunomodulating therapy, including cyclosporin, IgG-containing products, interleukins, interferons, systemic glucocorticosteroids, or exposure to an experimental HIV vaccine within 6 months prior to study entry.

4.2.10 Receipt of any vaccine within 30 days prior to study entry.

4.2.11 Allergy/sensitivity to study vaccine products, including adhesives, will be excluded.

4.2.12 Active drug or alcohol use or dependence that, in the opinion of the investigator, would interfere with adherence to study requirements.

4.2.13 Serious illness until subject either completes therapy or is clinically stable on therapy, in the opinion of the site investigator, for at least 14 days prior to study entry.

4.2.14 Hepatitis B surface antigen and/or anti-hepatitis C positive.

4.3 Study Enrollment Procedures

4.3.1 Prior to implementation of this protocol, the protocol and consent form must be approved by the local institutional ethics committee.

Once a candidate for study entry has been identified, details will be carefully discussed with the subject. The subject (or parent or legal guardian if the subject is under guardianship) will be asked to read and sign the consent form.

Only subjects who meet the eligibility criteria at both the screening and pre-entry visits will be enrolled. Cohorts will enroll serially. After all 3 subjects of a given cohort (#1 or #2) have received their study vaccine administrations, and have remained on study through at least the 14th day, if no subject in any cohort has experienced a Primary Safety endpoint (defined in section 9.2.1), then the study will dose escalate by opening enrollment of subjects into the next cohort.

5.0 STUDY TREATMENT

5.1 Regimens, Administration, and Duration

Study treatment is DermaVir.

5.1.1 Subjects who meet the inclusion criteria will be sequentially enrolled into each cohort:

- Cohort 1:
  - Three subjects will receive a single low-dose vaccination (0.1 mg DNA/subject, 0.8 mL total, administered over two skin sites of 80 cm^2 each, 0.4 mL/site) at study day 0.

- Cohort 2:
  - Three subjects will receive a medium-dose vaccination (0.4 mg DNA/subject, 3.2 mL total, administered over four skin sites of 80 cm^2 each, 0.8 mL/site) at study day 0.

- Cohort 3:
  - Three subjects will receive a high-dose vaccination (0.8 mg DNA/subject, 6.4 mL total, administered over eight skin sites of 80 cm^2 each, 0.8 mL/site) at study day 0.

5.1.2 LC0002 administration

a) Materials for administrationof LC002 will be supplied as a *DermaPrep* kit containing the following in a single pouch:
   - One yellow exfoliating sponge
   - Four stripping tapes
   - Two skin patches (each for vaccination of an 80 cm^2 area)

   In addition, the following materials are required:
   - Disposable razors
   - Surgical markers
   - Alcohol swabs
   - 1-ml graduated syringes
   - Needles (21-gauge, 1 1/2" length)

b) The final LC002 product should be kept at 4 °C after formulation and administered to the subject within 3 hours as follows (NOTE: Each step of the administration procedure must be documented on the CRF):

1. The subject should be seated comfortably on an examination table.

2. Identify the appropriate vaccination sites. Each site to be prepared corresponds to the size of one large DermaPrep patch.

	UPPER BACK				VENTRAL UPPER THIGH			
	Right lateral	Right median	Left median	Left lateral	Right lateral	Right median	Left median	Left lateral
Cohort 1	1	-	-	-	-	-	1	-
Cohort 2	1	-	-	1	-	1	1	-
Cohort 3	1	1	1	1	1	1	1	1

<u>**Note:**</u> It is advisable to proceed by preparing one site at a time.

3. Without removing the backing, hold the skin patch (Cohort 1&2: 1 patch at each site; Cohort 3: 2 patches next to each other at each site) to the vaccination site and use a surgical marker to demarcate approximately 3cm from the outer corners of the patch. This will serve as a guide for preparing the skin for vaccination.

4. Carefully shave the entire marked skin site, plus the additional 3cm margin, using a disposable razor.

5. Disinfect the entire marked skin site, plus the additional 3cm margin, using alcohol swab and wait for the skin to dry. Repeat this procedure using a fresh alcohol swab.
   <u>**Note:**</u> It is important that this procedure is performed thoroughly as it is the first step in removing the oily layer normally found on the surface of the skin. In addition, it improves adherence of the patch to the skin and thereby reduces the risk of leakage after vaccine application.

6. Gently exfoliate the entire marked skin site by rubbing the yellow exfoliating sponge 50 times back and forth over the demarcated area, applying light pressure, but taking care not to break the skin (1 exfoliation is equal to 1 forward and backward rub).

7. Apply one stripping tape to the site and immediately strip off in one quick movement to remove residual cell matter from exfoliation on the skin surface. Repeat if necessary, using same piece of stripping tape, in order to cover whole area of the site.

8. Repeat taping procedure with second stripping tape at a 90° angle to the first taping.

9. Apply the one skin patch to the site.
   The skin patch consists of 4 pieces: small white triangle backing (A), large white backing (B), semi-occlusive patch- thin, clear adhesive layer with a non-adhesive window, applied to skin (C), and a stabilizing layer - thick, clear adhesive layer, removed after application to skin (D).

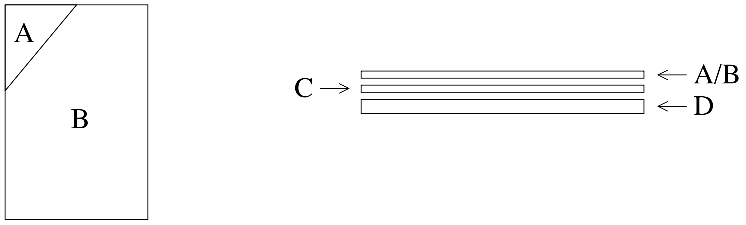

   a) Holding onto (A), remove only the large backing (B).

   b) Apply patch to the skin with (A) at the top. Ensure patch is placed within the demarcated and prepared skin site. Once adhered to the skin, a pocket is created between the patch and the skin surface. (A) remains attached to the patch, preventing the entire patch from sticking to the skin and allowing access to the pocket.

   c) Firmly press and smooth the entire perimeter of patch to ensure complete contact with the skin. Repeat this procedure several times to prevent leaking once vaccine is applied to the pocket.

   d) Remove the outer clear stabilizing layer (D), being careful not to detach the semi-occlusive patch (C) from the skin. Once the patch has been applied to the skin, (D) should start to peel away from the patch. Again firmly press and smooth edges and corners of the adhesive patch to ensure complete contact with the skin.

   e) Run a flat hand from bottom to top of the patch to squeeze air out of pocket.

10. Using the 1ml-syringe and needle, draw the volume of DermaVir required for one site from the vial(s) provided:
    - <u>Cohort 1 - Low-dose</u> vaccination: For each site, draw <u>0.4 ml</u> of LC002 into syringe.
    - <u>Cohort 2 - Medium-dose</u> vaccination: For each site, draw <u>0.8 ml</u> of LC002 from one vial into the syringe.
    - <u>Cohort 3 - High-dose</u> vaccination: For each site, draw <u>0.8 ml</u> of LC002 from one vial into the syringe.

<u>Note</u>: Discard needle into a sharps container before proceeding to next step.

11. Carefully insert the 1ml-sryinge (without needle), via the open corner formed by (A), into the pocket formed by the patch, so that the tip reaches the lower third of the pocket.

12. Slowly expel the DermaVir formulation into the pocket, and withdraw the syringe.

13. Remove (A) and close the pocket completely by sealing the upper edges of the patch, and check that all edges of the patch adhere well and are sealed.

14. Repeat this procedure for all remaining skin sites.
<u>**Note:**</u> Use a new syringe and needle to draw DermaVir for each site.

15. The skin patch will be removed after 3 hours **<u>at the clinical site</u>** by the investigator. Patches may be discarded with general waste but must be kept safely out of reach of children or animals.
<u>**Note:**</u> The investigator will evaluate local reactogenicity immediately after removal of the patch.

16. After removal of the skin patch, all treated sites are to be washed with clean water.

5.2 <u>Product Formulation and Preparation</u>

The individual components of DermaVir-LC002 will be supplied in vials as follows:

Cohort 1

Component	Content	Concentration	Volume	Appearance
VIAL 1	Plasmid DNA – pLWXu1	1 mg/mL	0.125 mL	Clear solution
VIAL 2	PEIm	13.6 mM	0.150 mL	Clear solution
AMPUL	Dextrose solution, USP	10%	5 mL	Clear solution

Cohort 2 and 3

Component	Content	Concentration	Volume	Appearance
VIAL 1*	Plasmid DNA – pLWXu1	1 mg/mL	0.425 mL	Clear solution
VIAL 2*	PEIm	13.6 mM	0.450 mL	Clear solution
AMPUL	Dextrose solution, USP	10%	5 mL	Clear solution

* **Cohort 3:** Two vials each of DNA and PEIm will be provided for formulation of the high dose.

5.2.1 Source and Storage Conditions of Components

Component	Manufacturer	Storage
Plasmid DNA – pLWXu1	Althea Technologies, Inc, San Diego, CA 92121, USA	-80°C, when possible; -20°C otherwise
PEIm	PolyPlus-transfection™, University of Strasbourg, School of Pharmacy Illkirch, Strasbourg, France	-80°C, when possible; -20°C otherwise
Dextrose solution, USP	Abbott Laboratories, North Chicago, IL 60064, USA	15 - 30°C
LC002	Trained personnel at clinical site	4 °C

5.2.2 Other materials required for formulation

- 1-ml graduated syringes (2)
- Needles (21-gauge, 1 1/2” length) for formulation (2)

5.2.3 Procedures

This procedure is to be performed only by specifically trained individuals and should take place under aseptic conditions, such as in a pharmaceutical hood or laminar flow hood.

The following final manufacturing steps of LC002 should be completed within 3 hours of LC002 application. The DermaVir-LC002 Formulation Record (Appendix V), is to be filled out in the CRF when following this procedure.

1. Thaw frozen components for 30 minutes at room temperature.

2. Use a syringe and needle to transfer the dextrose solution to **Vial 1** (DNA):
   - Cohort 1: transfer 0.375 mL
   - Cohort 2: transfer 1.275 mL
   - Cohort 3: transfer 1.275 mL.

3. Use the same syringe and needle to transfer the dextrose solution to **Vial 2** (PEIm).
   - Cohort 1: transfer 0.450 mL
   - Cohort 2: transfer 1.350 mL
   - Cohort 3: transfer 1.350 mL

   Discard syringe and needle after this step.

4. Both vials are inverted 10 times and then shaken down with a quick flick of the wrist so that as much of the solution as possible can be collected to the bottom of the vial and is not lost in the top.

5. Use a new syringe and needle to remove solution from **Vial 2** (PEIm):
   - Cohort 1: remove 0.50 mL
   - Cohort 2: remove 1.70 mL
   - Cohort 3: remove 1.70 mL

   and add this volume to **Vial 1** (DNA). Discard syringe and needle.
   Note: It is important to add Vial 2 to Vial 1, and NOT Vial 1 to Vial 2, for optimal formation of the PEIm/DNA complexes.

6. **Vial 1** containing all components is inverted 10 times and shaken down with a quick flick of the wrist. The DermaVir product is then ready for administration.

7. Fill out the DermaVir-LC002 Formulation Record with the amounts and batch numbers of each component used and the time of formulation.

8. Label final product vial with the appropriate label stating the Formulated Batch # and time of expiration (3 hours after formulation).

9. Batch #'s are determined as follows:

Date of formulation	Dash	Sequence of formulation for that day
MMDDYY	-	XX

   Example:
   Batch # for the second formulation performed on January 5, 2004: 010504-02.

10. Immediately deliver vial along with Form #: OPF-2008 to the clinical site.

11. The final DermaVir product should be kept at 4°C and administered to the subject within 3 hours or formulation.

Note: The volume of DermaVir formulated here is sufficient for a single dose (Cohorts 1 and 2, low and medium dose, respectively). Cohort 3 (high dose) requires two separate formulations to produce the volume of DermaVir required for a single dose.

Quantitative composition of the final dosage:

Dose	PEIm (13.6 mM, aqueous solution)*		DNA (1mg/mL, aqueous solution)		Dextrose (100 mg/mL, aqueous solution)		Dose of LC002	Treated skin area
	Volume (ml)	Quantity (mg)	Volume (ml)	Quantity (mg)	Volume (ml)	Quantity (mg)		
**Low**	0.1	0.000069	0.1	0.1	0.6	60	2 x 0.4 ml	2 x 80 cm^2
**Medium**	0.4	0.000276	0.4	0.4	2.4	240	4 x 0.8 ml	4 x 80 cm^2
**High**	0.8	0.000552	0.8	0.8	4.8	480	8 x 0.8 ml	8 x 80 cm^2

*This concentration is calculated based on the monomer of ethylenimine and 5% mannosilation (MW= 42 (MW of EI) x 180 (MW of mannose) x 0.05 (5% mannose) = 51. Therefore, 0.1 ml 13.6 mM PEIm has 69 ng PEIm.

5.3 Product Supply, Distribution, and Pharmacy

5.3.1 Study Product Acquisition

LC002-DermaVir will be provided by the Sponsor.

Antiretrovirals will not be supplied by the study and will need to be obtained by the subject.

5.4 Concomitant Medications

5.4.1 Required Medications

Subjects will be required to be on a stable regimen of HAART (containing drugs of at least two different classes), and remain on it while on study, that suppresses plasma HIV-1 RNA to < 50 copies/mL.

5.4.2 Prohibited Medications

The concomitant use of the following immunomodulatory therapies, which have potential putative effects on immunologic and/or virologic indices, are prohibited while on study:

- Systemic (IV and po) corticosteroids
- Thalidomide
- Cyclosporin
- Interferons
- Interleukins
- IgG-containing products

- Cimetidine (Tagamet)
- Acetylcysteine (NAC)
- Sargramostim (GM-CSF)
- Dinitrochlorobenzene (DNCB)
- Thymosin alpha 1 (thymosin alpha)
- Thymopentin
- Inosiplex (Isoprinosine)
- Polyribonucleoside (Ampligen)
- Ditiocarb sodium (Imuthiol)
- Experimental HIV vaccines

5.4.3 Precautionary Medications

- Treatment with topical corticosteroids is allowed except in close proximity to the vaccination sites in the 2 weeks prior to each vaccination.

- Use of local skin treatments is allowed, except in the targeted vaccination sites within 7 days of the vaccination. During this period, subjects should avoid manipulation of vaccinated areas to enable monitoring of possible vaccine related toxicity. Sites may be rinsed with water, but soap and other possible irritants should be avoided. In the case of discomfort at the vaccination site, cold packs may be administered as needed. All other local treatments should only be administered after consultation with the investigator.

5.4.4. Prohibited Activities

- Exposure to the sun (sunbathing, tanning bed) in the 2 weeks prior to vaccination.

- Laser hair removal at vaccination sites.

6.0 CLINICAL AND LABORATORY EVALUATIONS

The following clinical and laboratory evaluations will be performed at scheduled intervals as indicated below. A schema is provided in Section 6.1. Definitions and special instructions related to these evaluations are provided in Sections 6.2 and 6.3.

1. **Screening evaluations – to be performed within three months of entry:**
   Signed informed consent, documentation of HIV-1 infection, medical history, medication history, nadir CD4 count, complete physical examination (including signs and symptoms, diagnoses, Karnofsky score, height, weight, skin and lymph node status, and vital signs). Laboratory: hematology, blood chemistries, liver function tests, autoantibodies (anti-ds-DNA, anti-nuclear antibody), urinalysis, urine pregnancy test, hepatitis B and C screen (hepatitis B surface antigen and anti-hepatitis C antibodies), HIV-1 RNA, CD4/CD8 count and CD45RA/RO.

2. **Pre-entry evaluations – to be performed within 14 days of entry:**
   Laboratory: hematology, blood chemistries, liver function tests, urinalysis, HIV-1 RNA,

3. **Treatment-phase – evaluations from Day 0 to Day 28:**
   a. Medication history at Days 0, 28.
   b. Targeted physical examination at Days 0, 7, 14, 28.
   c. Complete physical examination at Day 28.
   d. Vaccine local site evaluations at Days 0 (immediately after removal of skin patch), 7, 14, 28.
   e. Hematology evaluations at Days 0, 7, 14, 28.
   f. Blood chemistries and liver function tests at Days 0, 7, 14, 28.
   g. Autoantibodies (ANA, anti-ds-DNA) at Day 28.
   h. Urinalysis at Days 0, 7, 14, 28.
   i. Urine pregnancy test at Day 0.
   j. HIV-1 RNA at Days 0, 7, 28.
   k. CD4/CD8 count and CD45RA/RO at Days 0, 7, 14, 28.
   l. Virus-specific immune responses: samples at Days 0, 14, 28.
   m. Plasma and PBMCs for storage at Days 0, 7, 14, 28.

4. **Post-treatment phase – evaluations after Day 28 (safety follow up):**
   a. Medication history at Weeks 12, 24, 36, 48.
   b. Targeted physical examination at Weeks 12, 24, and 36.
   c. Complete physical examination at Week 48.
   d. Hematology evaluations at Weeks 12, 24, 36 and 48.
   e. Blood chemistries and liver function tests at Weeks 12, 24, 36 and 48.
   f. Autoantibodies (ANA, anti-ds-DNA) at Week 48.
   g. Urinalysis at Weeks 12, 24, 36 and 48.
   h. HIV-1 RNA at Weeks 12, 24, 36 and 48.
   i. CD4/CD8 count at Weeks 12, 24, 36 and 48.

j. Plasma and PBMCs for storage at Weeks 12, 24, 36 and 48.

6.1 Schedule of Events

Evaluations	Screening		Treatment phase				Safety follow-up	
	Screening	Pre-entry	Day 0	Day 7	Day 14	Day 28	Weeks 12,24,36	Week 48
Signed Informed Consent	X							
Documentation of HIV	X							
Medical History	X							
Medication History	X		X			X	X	X
Nadir CD4	X							
Complete Physical Exam	X					X		X
Targeted Physical Exam			X	X	X	X	X	
LC002 Administration			**X**					
Vaccine Site Local Evaluation			X	X	X	X		
Hematology	X	X	X	X	X	X	X	X
Blood Chemistries	X	X	X	X	X	X	X	X
Liver Function Tests	X	X	X	X	X	X	X	X
Autoantibody Test	X					X		X
Urinalysis	X	X	X	X	X	X	X	X
Urine Pregnancy Test	X		X					
Hepatitis B and C Screen	X							
HIV-1 RNA	X	X	X	X		X	X	X
CD4/CD8 Count and CD45 RA/RO	X	X	X	X	X	X	X	X
Virus Specific Immune Response Assay			X		X	X		
Stored plasma and PBMC		X	X	X	X	X	X	X

6.2 Definitions for Schedule of Events

6.2.1 Screening Evaluations

Screening evaluations must occur prior to the subject taking any treatments.

Screening
Screening evaluations to determine eligibility must be completed within **3 months** prior to study entry.

Pre-entry
Pre-entry evaluations must be completed at least 24 hours after the screening evaluations and within 14 days prior to study entry.

6.2.2 On-Study Evaluations

Entry
Evaluations should occur after enrollment prior to vaccine administration and at least 24 hours after the pre-entry evaluations.

All subjects will visit the clinic on the weeks ± 3 days indicated in the Schedule of Events. Vaccine administration has to occur on the weeks indicated in the Schedule of Events ± 0 days.

6.2.3 Evaluations for **eligible** subjects who do not start study treatment

No further evaluations are required.

Subjects passing the screening and/or pre-entry evaluations who, for whatever reason, do not enter the study (Day 0) will not be considered as premature discontinuations.

6.2.5 Final Study Evaluations

Week 4 will be the final visit in the study treatment phase. Safety follow up continues to 48 weeks after immunization.

6.2.6 Pregnancy

All female subjects of child-bearing potential must have a negative urine pregnancy test on the day of vaccination. The subject should NOT receive immunizations until the result of the pregnancy test is known to be negative.

6.3 Special Instructions and Definitions of Evaluations

6.3.1 Documentation of HIV

HIV-1 infection, as documented by any licensed ELISA test kit and confirmed by Western blot, HIV-1 culture, HIV-1 antigen, plasma HIV-1 RNA, or a second antibody test by a method other than ELISA is acceptable as an alternative confirmatory test at any time prior to study entry.

6.3.2 Medical History

A medical history must be present in source documents and case report forms (CRFs). The medical history should include any previous HIV-related diagnoses and non-HIV-related diagnoses of major organ systems.

Any allergies to any medications and their formulations must be documented.

6.3.3. Medication History

Please note that the medications are to be recorded on the CRFs only when noted.

A medication history must be present in source documents for:

- Complete HIV treatment history, including start and stop dates of any antiretroviral medication (estimated if the exact dates cannot be obtained), immune-based therapy, or HIV-related vaccines, including blinded study medications. All changes in HAART therapy must be documented in the CRF.

- ALL concomitant medications such as prophylactic antimicrobial medications, antifungal, antipyretics, analgesics, allergy medications, oral contraceptives, topical or inhalant corticosteroids will be recorded on the CRF (including doses and start and stop dates) during the course of the study.

- All prescription medications (in addition to those noted above) including those taken within 30 days prior to study entry, including actual or estimated start and stop dates must be recorded in the CRF.

- Nonprescription medications and alternative therapies such as dietary supplements, acupuncture, herbal therapies, and visualization techniques taken within 30 days prior to study entry must be recorded on the CRF. Include actual or estimated start and stop dates.

- History of drug allergy must be recorded on the CRF.

6.3.4 Nadir (lowest) CD4 cell count

The subject's prior nadir CD4 cell count (absolute value and date) should be documented when possible with a copy of the nadir CD4 cell count report and recorded on CRF. If this documentation is not available, then subject recollection will suffice. For subjects who do not know the exact nadir value and for whom there is no source documentation, then recall of the categorical nadir (e.g., <50, 51-100, 101-200, 201-500, >500 cells/mm^3) will suffice.

6.3.5 Complete physical exam

Complete physical examination includes signs and symptoms, diagnoses, Karnofsky score, height, weight, skin and lymph node status, and vital signs (including seated blood pressure, pulse, respirations and oral temperature).

6.3.6 Targeted physical exam

A targeted physical examination including the vital signs (seated blood pressure, pulse, respirations, and oral temperature) must be conducted at study entry and all subsequent visits, to be driven by any signs and symptoms and diagnoses previously identified and any new signs and symptoms or diagnoses that the subject has experienced since the last visit.

<u>Signs and Symptoms</u>

At study entry, all signs/symptoms must be recorded. On study, any signs or symptoms that led to a change in treatment, regardless of grade, must be recorded in the CRFs. Record all grades of local reactions and any other signs or symptoms Grade 2 or higher in the CRFs.

All signs, symptoms, HIV-related and AIDS-defining events, deaths, and

toxicities must be documented, and must be recorded in the CRFs within 48 hours throughout the course of the study.

Refer to the Division of AIDS Table for Grading Adult Adverse Experiences for toxicity grading.

Diagnoses

All confirmed and probable diagnoses and any clinical events made since the last visit must be recorded in the source document and the CRF.

6.3.7 LC002 administration/evaluation

The first vaccine site evaluation (see section 7.1.1) will be performed immediately after removal of the skin patch (3h post application). Further vaccine site evaluation must occur on the days indicated in the Schedule of Events.

LABORATORY EVALUATIONS

Any laboratory toxicities that led to a change in treatment, regardless of grade, must be recorded in the CRFs. At baseline, record all laboratory values. For post-baseline assessments, all laboratory values must be documented in the subject's record, but only laboratory values Grade > 2 must be recorded in the CRFs.

All Grade ≥ 2 laboratory results must be recorded on the CRFs within 48 hours throughout the course of the study.

Refer to the Division of AIDS Table for Grading Adult Adverse Experiences (see Appendix IV) for toxicity grading.

6.3.9 Hematology

Hemoglobin, hematocrit, white blood cell count (WBC), absolute neutrophil count (ANC) and platelets.

6.3.10 Blood Chemistries

Albumin, BUN, electrolytes, creatinine.

6.3.11 Liver Function Tests

ALT, AST, alkaline phosphatase, total bilirubin.

6.3.12 Autoantibody Test

Anti-nuclear antibody (ANA), anti-double-stranded DNA antibody (anti-ds-DNA)

6.3.13 Urinalysis

Urinalysis (dipstick).

6.3.14 Urine pregnancy testing

For women with reproductive potential: a urine β-HCG test with a sensitivity of 25-50 mIU/mL. The pregnancy test must be negative before vaccine administration.

6.3.15 Hepatitis B and C screen

Hepatitis B surface antigen and anti-hepatitis C antibodies, to be performed locally.

6.3.16 Plasma HIV-1 RNA

The screening HIV-1 RNA must have been performed locally within three months of entry. Eligibility will be determined based on the screening value. The pre-entry HIV-1 RNA must be performed within 14 days prior to study entry. The baseline value will be the geometric mean of the pre-entry and entry determination. All tests will be performed locally.

Additional laboratory evaluations (not considered in the Division of AIDS Table for Grading Adult Adverse Experiences):
Red blood cell count (RBC), granulocytes, lymphocytes, monocytes, eosinophils, basophils, prothrombin time, creatinine, amylase, LDH, glucose, total protein, cholesterol and triglycerides, C-reactive protein, direct bilirubin, albumin.

IMMUNOLOGIC STUDIES

6.3.17 CD4/CD8

Obtain absolute CD4/CD8 count and percentages within 14 days prior to study entry from a laboratory. Eligibility for study participation will be determined by the screening measurement, which has to be obtained within three months of entry. A mean of the pre-entry and entry measurements will be used as the baseline value.

Evaluations for CD4+ and CD8+ counts and subset percentage evaluations should be performed at the same laboratory, if possible, for baseline calculation and throughout the course of the study.

Because of the diurnal variation in CD4+ and CD8+ cell counts, determinations for individual subjects should be obtained consistently in either the morning or the afternoon throughout the study, if possible.

NOTE: Each time a CD4/CD8 measurement is obtained, the local laboratory must perform a WBC and differential from a sample obtained at the same time.

Additional cell surface markers (CD45RA, CD45RO) will be evaluated to determine naïve/memory phenotype of CD4 and CD8 T cells.

6.3.18 Virus Specific Immune Response Assays

a) Quantification of HIV-specific CD3+/CD8+ T cells by flow cytometry using intracellular cytokine (IFN-gamma) staining (1). (VIR Assay, see Appendix III for description).
b) HIV-specific proliferative responses (LPA, see Appendix III)
c) Quantification of HIV-peptide pool induced cytokine expressing cells (ELISPOT, ICC, see Appendix III)

NOTE: The immunological assays under b) and c) will be performed by an independent immunological laboratory (Prof. B. Autran, Laboratoire d'Immunologie Cellulaire et Tissulaire, Hôpital Pitié-Salpêtrière, Paris, France).

6.3.19 Stored Plasma and PBMC

Plasma and PBMC will be stored according to Appendix III.

6.4 Off-Drug Requirements

Additional 12-week period safety monitoring and reporting of serious adverse experiences (SAEs) continues to be required upon completion or discontinuation of study treatment regardless of whether a protocol follow-up period is scheduled to occur. After 12 weeks OFF study treatment, there are four types of events that must be reported if the relationship to the study drug is assessed by the site physician as definitely, possibly, or unable to judge: DEATHS, NEW ONSET CANCERS, CONGENITAL ANOMALIES, AND PERMANENT DISABILITIES.

7.0 TOXICITY MANAGEMENT

7.1 Reactions Thought Definitely, Possibly, or Probably Related to Study Vaccine

7.1.1 Local reactions

Local reactions will be graded using the local reaction assessment scale (Table 1).

Table 1: Local reaction assessment

Grade	Description
0 = None	N/A
1 = Mild	Macular or papular eruption, erythema or induration that is asymptomatic or mildly symptomatic
2 = Moderate	Macular or papular eruption, erythema or induration with pruritus or other associated moderate symptoms
3= Severe	Ulceration, blistering, superinfection or phlebitis
4= Potentially Life-threatening	Necrosis of the skin

7.1.2 Systemic reactions

Systemic reactions will be graded according to the Division of AIDS Table for Grading Adult Adverse Experiences (see Appendix IV). The Principal Investigator should be contacted within 48 hours for any non-local Grade 3 or 4 reactions (for example, elevated temperatures following immunization) thought definitely, possibly, or probably related to vaccination with LC002.

7.2 Antiretroviral Drugs

Unanticipated and anticipated toxicities from the HAART regimen will be graded according to the Division of AIDS Table for Grading Adult Adverse Experiences (see Appendix IV). Any Grade 3 or 4 toxicities that result in a temporary or permanent change in an antiretroviral therapy must be reported to the Principal Investigator.

Anticipated toxicities resulting from components of the HAART regimen will be managed by the subject's clinician according to best clinical practice including dose reductions when indicated.

If the HIV-1 viral load becomes detectable (plasma HIV-1 RNA > 1,000 copies/mL) either during the treatment phase (Day 0 to 28) or 48-week follow up, it should be repeated one week later and compliance issues should be ruled out. If the repeated HIV-1 viral load is detectable (plasma HIV-1 RNA > 1,000 copies/ml) genotyping should be performed and HAART changed if necessary.

In case of virologic failure, genotyping will also be performed on the nef gene. As the nef gene contained in the DNA of LC002 is truncated, detection of a truncated nef gene would indicate recombination of the patient’s virus with the vaccine. All patients with virologic failure will be followed for the complete 48-week follow up period.

8.0 CRITERIA FOR TREATMENT DISCONTINUATION

- Vaccine-related toxicity (see section 7.0 Toxicity Management).
- Requirement of prohibited concomitant medications (see section 5.4)
- Failure by the subject to attend 2 consecutive clinic visits.
- Pregnancy or breast-feeding.
- Request by the subject to withdraw.
- Request of the primary care provider if s/he thinks the study is no longer in the best interest of the subject.
- Subject judged by the investigator to be at significant risk of failing to comply with the provisions of the protocol as to cause harm to self or seriously interfere with the validity of the study results.
- At the discretion of the investigator or sponsor.

9.0 STATISTICAL CONSIDERATIONS

9.1 General Design Issues

GIHU004 is a phase I sequential dose escalation cohort study designed to select a tolerable dosing regimen for the DermaVir vaccine and to explore its immunogenicity in the study population. Three cohorts of 9 subjects each will be sequentially enrolled (depending on safety outcomes, which determine whether to dose escalate or not).

9.1.1 Primary Objectives

To evaluate the safety and tolerability of three different doses of LC002 in HIV-infected patients on HAART.

9.1.2 Secondary Objectives

To explore the immunogenicity of the LC002 for the treatment of individuals with chronic HIV-1 infection and who have HAART-induced durable suppression of viral replication.

9.1.3 Dose Escalation Rule

Dose escalation will occur if

1) at least 2 subjects were on study until at least 14 days after receiving their study vaccination and
2) no subject in the current or lower dose cohort experienced a Primary Safety Endpoint.

9.2 Endpoints

9.2.1 Primary endpoint: Safety

Occurrence of at least one grade 3 or higher adverse event including signs/symptoms, lab toxicities and clinical events that is "possibly, probably," or "definitely" related to study treatment (as judged by the Principal Investigator, including site clinicians on the team) any time from the first day of study treatment until 28 days after LC002 administration.

9.2.2 Secondary endpoints

The following endpoints will be observed at all on-study scheduled evaluation days (section 6.1).

9.2.2.1 Change in CD4+ T-Cell count/mL in PBMC from baseline values.

9.2.2.2 Change in CD8+ T-Cell count/mL in PBMC from baseline values.

9.2.2.3 Total HIV-specific lymphocyte count and percent (CD3+ T-cells) based on a flow cytometric assay to detect antigen-specific IFN-gamma-producing cells that respond to whole Zn-finger-inactivated virus stimulation: To measure changes in the magnitude of HIV-specific CD3+ immune responses in the blood induced by vaccination.

9.2.2.4. HIV-specific CD8+ T-cell count and percent (CD3+/CD8+) based on a flow cytometric assay to detect antigen-specific IFN-gamma-producing cells that respond to whole Zn-finger-inactivated virus stimulation:
To measure changes in the magnitude of HIV-specific CD8+ immune responses in the blood induced by vaccination.

9.2.2.5. Tolerability: occurrence of premature study treatment discontinuation (i.e. less than three hours of patch application at all designated sites) due to subject/parent/guardian/physician requesting discontinuation even though no protocol-defined toxicity endpoint had been reached (e.g. grade 1 or 2 toxicity or refuses rechallenge for higher grade, if appropriate.)

9.2.2.5. To evaluate if anti-DNA antibody responses develop after immunization.

9.2.2.6 Whether HIV-1 RNA copies/mL is below 50 or not.

9.3 Accrual

Any subject who withdraws from the study prematurely without experiencing a primary safety endpoint before Week 4 evaluation, and whose reasons for withdrawing from the study are unrelated to any real or perceived effect of the study vaccination or its administration, will be replaced with a subject assigned to the same dose cohort and the same arm. If possible, all subjects who discontinue the study prematurely will be followed for 48 weeks for study treatment safety information. A maximum of two subjects per cohort will be replaced.

10.0 DATA COLLECTION AND MONITORING AND ADVERSE EXPERIENCE REPORTING

10.1 Records To Be Kept

Case report forms (CRF) will be provided for each subject. Subjects must not be identified by name on any CRFs. Subjects will be identified by the Patient Identification Number (PID) and Study Identification Number (SID) provided by the CRO data management department upon registration.

10.2 Role of Data Management

10.2.1 Instructions concerning the recording of study data on CRFs will be provided by the CRO.

10.2.2 It is the responsibility of the CRO to assure the quality of computerized data for this study. This role extends from collecting the CRF to generation of the final study databases and evaluation of data.

10.3 Clinical Site Monitoring and Record Availability

10.3.1 Site monitors under contract to the CRO will visit the clinical research site to review the individual subject records, including consent forms, CRFs, supporting data, laboratory specimen records, and medical records (physicians' progress notes, nurses' notes, individuals' hospital charts), to ensure protection of study subjects, compliance with the protocol, and accuracy and completeness of records. The monitors also will inspect sites' regulatory files to ensure that regulatory requirements are being followed and site's pharmacies to review product storage and management.

10.3.2 The investigator will make study documents (e.g., consent forms, drug distribution forms, CRFs) and pertinent hospital or clinic records readily available for inspection by the local Ethical Committee, the site monitors, the National Institute for Pharmacy (NIP), or the sponsor's designee for confirmation of the study data.

10.4 Serious Adverse Experience (SAE) Reporting

This protocol follows intensive reporting requirements.

Serious adverse experiences must be reported to the National Institute of Pharmacy and Sponsor's representative in Hungary within 24 hours by telephone. In addition, SAE must be reported to the NIP and sponsor within 3 days by completion of the standard EU SAE reporting form.

NIP/OGYI contact:	Tel: 317-14-88<br>Fax: 318-11-67
Sponsor's representative, Hungary:	Tel: 258-4078<br>Fax: 258-7416
Sponsor USA:	Tel: +1 202 338 9580<br>Fax: +1 202 338 9583

11.0 HUMAN SUBJECTS

11.1 Institutional Local Ethical Committee and Informed Consent

This protocol and the informed consent documents and any subsequent modifications will be reviewed and approved by the Local Ethics Committee responsible for oversight of the study. A signed consent form will be obtained from the subject (or parent, legal guardian, or person with power of attorney for subjects who cannot consent for themselves). The information file and consent form will describe the purpose of the study, the procedures to be followed, and the risks and benefits of participation. A copy of the consent form will be given to the subject, parent, or legal guardian, and this fact will be documented in the subject's record.

11.2 Subject Confidentiality

All laboratory specimens, evaluation forms, reports, and other records that leave the site will be identified by coded number only to maintain subject confidentiality. All records will be kept locked. All computer entry and networking programs will be done with coded numbers only. Clinical information will not be released without written permission of the subject, except as necessary for monitoring.

11.3 Study Discontinuation

The study may be discontinued at any time by the Local Ethical Committee, the Principal Investigator, the pharmaceutical sponsor(s), the NIP, or other government agencies as part of their duties to ensure that research subjects are protected.

12.0 PUBLICATION OF RESEARCH FINDINGS

Any presentation, abstract, or manuscript will be sent for approval to the Sponsor prior to submission.

13.0 BIOHAZARD CONTAINMENT

As the transmission of HIV and other blood-borne pathogens can occur through contact with contaminated needles, blood, and blood products, appropriate blood and secretion precautions will be employed by all personnel in the drawing of blood and shipping and handling of all specimens for this study, as currently recommended by National Institute of Epidemiology.

14.0 REFERENCES

1. Jianqing Xu, Whitman L, Lori F, Lisziewicz J. Quantification of HIV-specific CD8 T cells by in vitro stimulation with inactivated viral particles. AIDS 2002; 16: 1849-1857.

2. Hammer SM, Squires KE, Hughes MD, et al. A controlled trial of two nucleoside analogues plus indinavir in persons with human immunodeficiency virus infection and CD4 cell counts of 200 per cubic millimeter or less. N Engl J Med 1997; 337: 725-33.

3. Palella FJ Jr, Delaney KM, Moorman AC, Loveless MO, Fuhrer J, Satten GA, Aschman DJ, Holmberg SD. Declining morbidity and mortality among patients with advanced human immunodeficiency virus infection. HIV Outpatient Study Investigators. N Engl J Med 1998; 338: 853-60.

4. Finzi D, Blankson J, Siliciano JD, et al. Latent infection of CD4+ T cells provides a mechanism for lifelong persistence of HIV-1, even in patients on effective combination therapy. Nat Med 1999; 5: 512-17.

5. Chun TW, Fauci AS. Latent reservoirs of HIV: obstacles to the eradication of virus. Proc Natl Acad Sci USA 1999; 96: 10958-961.

6. Miedema F, Meyaard L, Koot M, et al. Changing virus-host interactions in the course of HIV-1 infection. Immunol Rev 1994; 140: 35-72.

7. Krowka IF, Stites DP, Jain S, et al. Lymphocyte proliferative responses to human immunodefIciency virus antigens in vitro. J Clin Invest 1989; 83: 1198-203.

8. Musey L, Hughes J, Schacker T, Shea T, Corey L, McElrath MI. Cytotoxic-T-cell responses, viral load, and disease progression in early human immunodeficiency virus type 1 infection. N Engl J Med 1997; 337: 1267-274.

9. Betts MR, Ambrozak DR, Douek DC, et al. Analysis of total human immunodeficiency virus (HIV)-specific CD4(+) and CD8(+) T-cell responses: Relationship to viral load in untreated HIV infection. J Virol 2001; 75:11983-91.

10. Abbas AK. Die and let live. Eliminating dangerous lymphocytes. Cell 1996; 84: 655-7.

11. Weissman D, Barker TD, Fauci AS. The efficiency of acute infection of CD4+ T cells is markedly enhanced in the setting of antigen-specific immune activation. J Exp Med 1996; 183: 687-92.

12. Fauci AS, Pantaleo G, Stanley S, Weissman D. Immuopathogenic mechanisms of HIV infection. Ann Intern Med 1996; 124: 654-63.

13. Champagne P, Ogg GS, King AS, et al. Skewed maturation of memory HIV-specific CD8 T lymphocytes. Nature 2001; 410: 106-111.

14. Pitcher Cl, Quittner C, Peterson DM, et al. HIV -1-specific CD4+ T cells are detectable in most individuals with active HIV-1 infection, but decline with prolonged viral suppression. Nat Med 1999; 5: 518-25.

15. Rosenberg ES, Eillingsley JM, Caliendo AM, et al. Vigorous HIV-1-specific CD4+ T cell responses associated with control of viremia. Science 1997; 278: 1447-50.

16. Cao Y, Qin L, Zhang L, Safrit l, Ho DD. Virologic and immunologic characterization of long-term survivors of human immunodeficiency virus type 1 infection. N Engl J Med 1995; 332: 201-08.

17. Pantaleo G, Menzo S, Vaccarezza M, et al. Studies in subjects with long-term nonprogressive human immunodeficiency virus infection. N Engl J Med 1995;332:209-16.

18. Goh WC, Markee J, Akridge RE, et al. Protection against human immunodeficiency virus type 1 infection in persons with repeated exposure: evidence for T cell immunity in the absence of inherited CCR5 coreceptor defects. J Infect Dis 1999; 179: 548-57.

19. Kaul R, Rowland-Jones SL, Kimani J, et al. Late seroconversion in HIV-resistant Nairobi prostitutes despite pre-existing HIV-specific CD8+ responses. J Clin Invest 2001; 107: 341-9.

20. Koup RA, Safrit JT, Cao Y, et al. Temporal association of cellular immune responses with the initial control of viremia in primary human immunodeficiency virus type 1 syndrome. J Virol 1994; 68: 4650-55.

21. Barouch DH, Santra S, Schmitz JE, et al. Control of viremia and prevention of clinical AIDS in rhesus monkeys by cytokine-augmented DNA vaccination. Science 2000; 290: 486-92.

22. Barouch DH, Kunstman J, Kuroda MJ, et al. Eventual AIDS vaccine failure in a rhesus monkey by viral escape from cytotoxic T lymphocytes. Nature 2002; 415: 335-9.

23. Schmitz JE, Kuroda MJ, Santra S, et al. Control of viremia in simian immunodeficiency virus infection by CD8+ lymphocytes. Science 1999; 283: 857-60.

24. Jin X, Bauer DE, Tuttleton SE, et al. Dramatic rise in plasma viremia after CD8(+) T cell depletion in simian immunodeficiency virus-infected macaques. J Exp Med 1999;189: 991-8.

25. Lisziewicz J, Xu J, Trocio J, Whitman L, Markham P, Arya S, et al. A novel topical DNA vaccine (DermaVir) for the induction of T cell immunity. [Abstract 286-W] 9th Conference on Retroviruses and Opportunistic Infections 2002.

26. Lisziewicz J, et al. Induction of potent human immunodeficiency virus type 1-specific T-cell-restricted immunity by genetically modified dendritic cells. J Virol 2001; 75 (16): 7621-28.

27. Lori F, et al. Control of SIV rebound through structured treatment interruptions during early infection. Science 2000; 290 (5496): 1591-93.

28. Lisziewicz J, et al. Control of HIV despite the discontinuation of antiretroviral therapy. N Engl J Med 1999; 340 (21): 1683-1684.

29. Rosenberg ES, et al. Immune control of HIV-1 after early treatment of acute infection. Nature 2000; 407 (6803): 523-6.

30. Rowland-Jones S, et al. HIV-specific cytotoxic T-cells in HIV-exposed but uninfected Gambian women. Nat Med 1995; 1(1): 59-64.

31. Almond N, et al. Protection by attenuated simian immunodeficiency virus in macaques against challenge with virus-infected cells. Lancet 1995; 345 (8961): 1342-44.

32. Cara A, et al. Self-limiting, cell-type dependent replication of an integrase defective HIV-1 in human macrophages but not in human T-lymphocytes. Virology 1995; 208: 646-50.

33. Stevenson M, et al. HIV-1 replication is controlled at the level of T cell activation and proviral integration. Embo J 1990; 9 (5): 1551-60.

34. Zufferey R. et al. Self-inactivating lentivirus vector for safe and efficient in vivo gene delivery. J Virol 1998; 72: 9873-9880.

35. McDonnell WM, Askari FK. DNA vaccines. N Engl J Med 1996; 334(1): 42-5.

36. Erbacher P, et al. Transfection and physical properties of various saccharide, poly(ethylene glycol), and antibody-derivatized polyethylenimines (PEI). J Gene Med 1999; 1(3): 210-22.

37. Boussif O, et al. A versatile vector for gene and oligonucleotide transfer into cells in culture and in vivo: polyethylenimine. Proc Natl Acad Sci U S A 1995; 92(16): 7297-7301.

38. Hunt P, Deeks SG, Rodríguez B, et al. Continued CD4+ T-cell gains in patients with 4 years of suppressed viral load on HAART. [abstract 480-M]. 9th Conference on Retrovirus and Opportunistic Infections. Seattle, February 2002.

39. Kovacs JA, Masur H. Prophylaxis against opportunistic infections in patients with human immunodeficiency virus infection. N Engl J Med 2000; 342: 1416-29.

40. USPHS/IDSA Prevention of Opportunistic Infections Working Group. 2001 USPHS/IDSA Guidelines for the prevention of opportunistic infections in persons infected with HIV. Clin Infect Dis 2000; 30: S29-S65.

41. Lederman MM, Connick E, Landay A, et al. Immunologic responses associated with 12 weeks of combination antiretroviral therapy consisting of zidovudine, lamivudine and ritonavir: Results of AIDS Clinical Trials Group Protocol 1315. J Infect Dis 1998; 178: 70-79.

42. Ogg GS, Jin X, Bonhoeffer S, et al. Decay kinetics of human immunodeficiency virus-specific effector cytotoxic T lymphocytes after combination antiretroviral therapy. J Virol 1999; 73: 797-800.

43. Kalams SA, Goulder PJ, Shea AK, Jones NG, Trocha AK, Ogg GS, Walker BD. Levels of human immunodeficiency virus type 1-specific cytotoxic T- lymphocyte effector and memory responses decline after suppression of viremia with highly active antiretroviral therapy. J Virol 1999; 73: 6721-28.

44. Pitcher CJ, Quittner C, Peterson DM, Connors M, Koup RA, Maino VC, Picker LJ. HIV-1-specific CD4+ T cells are detectable in most individuals with active HIV-1 infection, but decline with prolonged viral suppression. Nat Med 1999; 5: 518-25.

45. Eron JJ Jr., Ashby MA, Giordano MF, et al. Randomised trial of MNrgp120 HIV -1 vaccine in symptomless HIV -1 infection. Lancet 1996; 348: 1547-1551.

46. Valentine FT, Kundu S, Haslett PA, et al. A randomized, placebo-controlled study of the immunogenicity of human immunodeficiency virus (HIV) rgp160 vaccine in HIV-infected subjects with > or = 400/mm3 CD4 T lymphocytes (AIDS Clinical Trials Group Protocol 137). J Infect Dis 1996; 173: 1336-46.

47. Kundu SK, Katzenstein D, Valentine FT, Spino C, Efron B, Merigan TC. Effect of therapeutic immunization with recombinant gp160 HIV-1 vaccine on HIV-1 proviral DNA and plasma RNA: relationship to cellular immune responses. J Acquir Immune Defic Syndr Hum Retrovirol 1997; 15: 269-74.

48. Leandersson AC, Bratt G, Hinkula J, et al. Induction of specific T -cell responses in HIV infection. AIDS 1998; 12: 157-66.

49. Sandstrom E, Wahren B. Therapeutic immunisation with recombinant gp160 in HIV-1 infection: a randomised double-blind placebo-controlled trial. Nordic VAC-04 Study Group. Lancet 1999; 353: 1735-42.

50. Kelleher AD, Roggensack M, Jaramillo AB, et al. Safety and immunogenicity of a candidate therapeutic vaccine, p24 virus-like particle, combined with zidovudine, in asymptomatic subjects. Community HIV Research Network Investigators. AIDS 1998; 12: 175-82.

51. MacGregor RR, Boyer JD, Ugen KE, Lacy KE, et al. First human trial of a DNA-based vaccine for treatment of human immunodeficiency virus type 1 infection: safety and host response. J Infect Dis 1998; 178: 92-100.

52. Birx DL, Loomis-Price LD, Aronson N, et al. Efficacy testing of recombinant human immunodeficiency virus (HIV) gp160 as a therapeutic vaccine in early-stage HIV-1-infected volunteers. Rgp160 Phase II Vaccine Investigators. J Infect Dis 2000;181:881-9.

53. Schooley RT , Spino C, Kuritzkes D et al. Two double-blinded, randomized, comparative trials of 4 human immunodeficiency virus type 1 (HIV-1) envelope vaccines in HIV-1- infected individuals across a spectrum of disease severity: AIDS Clinical Trials Group 209 and 214. J Infect Dis 2000; 182: 1357 -64.

54. Kahn JO, Cherng DW, Mayer K, Murray H, Lagakos S. Evaluation of HIV-1 immunogen, an immunologic modifier, administered to patients infected with HIV having 300 to 549 x 106/L CD4 cell counts: A randomized controlled trial. JAMA 2000; 284: 2193-202.

55. Lisziewicz J, Rosenberg E, Lieberman J, et al. Control of HIV despite the discontinuation of antiretroviral therapy. N Engl J Med 1999; 340: 1683-84.

56. Rosenberg ES, Altfeld M, Poon SH, et al.. Immune control of HIV-1 after early treatment of acute infection. Nature 2000; 407: 523-26.

57. Hirschel B, Fagard C, Oxenius A, et al, for the Swiss HIV Cohort Study. SSITT: A prospective trial of treatment interruptions in HIV infection. [abstract 528-M]. 9th Conference on Retrovirus and Opportunistic Infections. Seattle, February 2002.

58. Banchereau J, Steinman RM. Dendritic cells and the control of immunity. Nature 1998; 392: 245-52.

59. Lanzavecchia A, Sallusto F. Dynamics of T lymphocyte responses: intermediates, effectors, and memory cells. Science 2000; 290: 92-7.

60. Bhardwaj N, Bender A, Gonzalez N, Bui LK, Garrett MC, Steinman RM. Stimulation of human anti-viral CD8+ cytolytic T lymphocytes by dendritic cells. Adv Exp Med Biol 1995; 378: 375-9.

61. Cella M, Salio M, Sakakibara Y, Julkunen I, Lanzavecchia A. Maturation, activation, and protection of dendritic cells induced by double-stranded RNA. J Exp Med 1999; 189: 821-9.

62. Ludewig B, Ehl S, Karrer U, Odematt B, Hengartner H, Zinkernagel RM. Dendritic cells efficiently induce protective antiviral immunity. J Virol 1998; 72: 3812-18.

63. Reinhardt RL, Khoruts A, Merica R, Zell T, Jenkins MK. Visualizing the generation of memory CD4 T cells in the whole body. Nature 2001; 410: 101-5.

64. Masopust D, Vezys V, Marzo AL, Lefrancois L. Preferential localization of effector memory cells in nonlymphoid tissue. Science 2001; 291: 2413-17.

65. von Andrian UH, Mackay CR. T-cell function and migration. Two sides of the same coin. N Engl J Med 2000; 343: 1020-34.

66. Kirk CJ, Mule JJ. Gene-modified dendritic cells for use in tumor vaccines. Hum Gene Ther 2000; 11: 797-806.

67. Condon C, Watkins SC, Celluzzi CM, Thompson K, Falo LD Jr. DNA-based immunization by in vivo transfection of dendritic cells. Nat Med 1996; 2: 1122-8.

68. Tuting T, Storkus WJ, Falo LD Jr. DNA immunization targeting the skin: molecular control of adaptive immunity. J Invest Dermatol 1998; 111: 183-8.

69. Lisziewicz J, Xu J, Trocio L, et al. Control of viral load rebound during treatment interruption in macaques with AIDS induced by a novel topical DNA immunization (DermaVir). [abstract 312-W] 9th Conference on Retroviruses and Opportunistic Infections. Seattle, February 2002.

70. Lori F, Lewis MG, Xu J, et al. Control of SIV rebound through structured treatment interruptions during early infection. Science 2000; 290: 1591-693.

71. Lisziewicz J, Xu J, Lewis M, Trocio J, Whitman L, Lori F. Safety, immunogenicity and antiviral efficacy of a new topical DNA vaccine in macaques with chronic infection and AIDS. [Abstract LbOr11] XIVth International AIDS Conference. Barcelona, Spain, 2002.

APPENDIX I: DEFINITION OF CDC CATEGORY C EVENTS

CDC category C events are defined in 1993 Revised Classification System for HIV Infection and Expanded Surveillance Case Definition for AIDS Among Adolescents and Adults (1):

- Candidiasis of bronchi, trachea, or lungs
- Candidiasis, esophageal
- Cervical cancer, invasive*
- Coccidioidomycosis, disseminated or extrapulmonary
- Cryptococcosis, extrapulmonary
- Cryptosporidiosis, chronic intestinal (greater than 1 month's duration)
- Cytomegalovirus disease (other than liver, spleen, or nodes)
- Cytomegalovirus retinitis (with loss of vision)
- Encephalopathy, HIV-related
- Herpes simplex: chronic ulcer(s) (greater than 1 month's duration); or bronchitis, pneumonitis, or esophagitis
- Histoplasmosis, disseminated or extrapulmonary
- Isosporiasis, chronic intestinal (greater than 1 month's duration)
- Kaposi's sarcoma
- Lymphoma, Burkitt's (or equivalent term)
- Lymphoma, immunoblastic (or equivalent term)
- Lymphoma, primary, of brain
- *Mycobacterium avium* complex or *M. kansasii*, disseminated or extrapulmonary
- *Mycobacterium tuberculosis*, any site (pulmonary* or extrapulmonary)

- Mycobacterium, other species or unidentified species, disseminated or extrapulmonary
- *Pneumocystis carinii* pneumonia
- Pneumonia, recurrent*
- Progressive multifocal leukoencephalopathy
- Salmonella septicemia, recurrent
- Toxoplasmosis of brain
- Wasting syndrome due to HIV

---

*Added in the 1993 expansion of the AIDS surveillance case definition.

APPENDIX II: KARNOFSKY PERFORMANCE SCORE

100%	Normal, no complaints; no evidence of disease
90%	Able to carry on normal activity; minor signs or symptoms of disease
80%	Normal activity with effort; some signs or symptoms of disease
70%	Cares for self; unable to carry on normal activity or do active work
60%	Requires occasional assistance but is able to care for most needs
50%	Requires considerable assistance and frequent medical care
40%	Disabled, requires special care and assistance
30%	Severely disabled; hospitalization is indicated though death is not imminent.
20%	Very sick; hospitalization is necessary
10%	Moribund, fatal processes progressing rapidly
0%	Dead

APPENDIX III: IMMUNOLOGY: SPECIMEN COLLECTION, ASSAY DESCRIPTION

1.0 Specimen Collection:

All specimens will be collected per section 6.1, Schedule of Events. Handling of all immunology specimens for this protocol is included in this appendix. The following tubes should be collected according to the Schedule of Events:

Tube Type/ Specimen	Assay	Aliquots	Send to	Lab Contact
2 x 10-mL ACD tubes	Virus Specific Immune Response assay	2 x 10-mL whole blood	Pr. Brigittte Autran<br>Laboratoire d'Immunologie Cellulaire et Tissulaire<br>INSERM U543<br>Hôpital Pitié-Salpêtrière<br>83 Bld de l'Hôpital<br>Bâtiment CERVI - 4ème étage<br>75013 PARIS, FRANCE<br>Tel: +33-1-42-17-74-81<br>Fax: +33-1-42-17-74-90	Prof. B. Autran
4 x sodium citrate cell preparation tubes (CPT)	Stored plasma and PBMC	Store 1 x 2-mL aliquot of plasma at –20 °C and freeze PMBCs in 3 x 1-mL vials in liquid N_2	Flow cytometric Laboratory<br>Bldg 22, Room 1-2	Mr. Zsolt Janosi

2.0 Virus-Specific Immune Response Assays

2.1 VIR assay

The Virus Specific Immune Response (VIR) assay will be used to evaluate the immunogenicity of the LC002 immunization of patients enrolled in the GIHU004 clinical study. This immune diagnostic test quantifies the HIV-specific T cells of a patient's immune system that may be engaged by the immune system to control HIV-1 during viral rebound. Therefore, the assay was designed to mimic in vitro HIV-1 viral rebound by adding viral particles to peripheral blood mononuclear cells (PBMC). Chemically inactivated HIV-1 with functional envelope glycoproteins is added in vitro to PBMC isolated from HIV-infected individuals. The inactivated particles can be taken up and processed by functional antigen-presenting cells (APC), which are present in bulk PBMC. These APC can then activate functional HIV-specific T cells to produce IFN-γ. IFN-γ is an early response marker that is produced immediately after antigen specific T cell activation in Th1-type responses. The IFN-γ producing cells are measurable by flow cytometry after Intracellular Cytokine (IC) staining and characterized by parallel staining with antibodies recognizing lymphocyte surface markers like CD3 and CD8. The VIR assay differs substantially from other IC based assays in that it relies on APC to internalize whole viral particles and present viral antigens to T lymphocytes. Therefore, all cells and all viral antigens involved in an immune response are represented in the

assay. The assay allows us to monitor the quantity of virus specific T cells quickly and easily. Furthermore, there is a tight correlation between conventional, functional bulk [^{51}Cr]-release (CTL) assay and the results of the VIR assay. The VIR assay can be used to characterize T cell-mediated immune responses in any patient population since it is not restricted by the HLA haplotype of the host.

2.1. LPA

A lymphocyte proliferation assay (LPA) will be used to evaluate the proliferative capacity of HIV specific CD4 T cells in response of stimulation with recombinant HIV peptides and proteins including p24. This assay planned to be performed on fresh cells.

2.2. Interferon-gamma ELISPOT

The ELISPOT assay will be used to enumerate cytokine producing HIV-specific CD8 responding to peptide stimulation. This technique is designed to determine the frequency of cytokine producing cells under HIV-specific stimulation, and the follow-up the frequency of HIV-specific T cells during clinical trials. Since lack of viral control occurs despite high frequencies of HIV-specific IFN-gamma-secreting CD8+ T cells and the recognition of multiple epitopes within virus proteins, this assay will be used only as indication for the frequency of HIV-specific cells.

2.3. ICC

ICC (intra-cellular flow cytometry) assay using intracellular IFN-gamma and IL-2 and surface CD4 and CD8 markers is planned to investigate the HIV-specific effector and memory cell pool in the periperial blood. Antigen-specific CD4 and CD8 T-cells are divided into three functionally distinct populations: cells that secrete IL-2 but not IFN-gamma, cells that secrete both IL-2 and IFN-gamma, and cells that secrete IFN-gamma but not IL-2. These functionally distinct cell populations are associated with different conditions of antigen persistence and antigen load. The single IL-2 response is typical of antigen clearance, the single IFN-gamma response is typical of antigen persistence and high antigen load, and the polyfunctional IL-2 plus IFN-gamma response is typical of protracted antigen exposure and low antigen load. In HIV infection there is a skewing of memory CD8+ T cells toward those that secrete IFN-gamma, whereas there is no evidence of virus-specific CD8+ T cells with the capacity to proliferate or to secrete IL-2. The presence of virus-specific CD8+ T cells that can proliferate and secrete IL-2 seems to be associated with low levels of antigen load and virus control, as in CMV and EBV infections and in HIV-1 infection in individuals with nonprogressive disease.

3.0 Plasma and PBMC will be isolated according the following Standard Operating Procedures (SOP) and Worksheet:

OP-2028: Procedure to Isolate and Store Plasma and PBMC from Whole Blood Collected in Cell Preparation Tubes with Sodium Citrate, GIHU004

OPF-2028: PMBC Isolation and Plasma Collection, GIHU004

APPENDIX IV: TOXICITY GRADING

DIVISION OF AIDS TABLE for GRADING SEVERITY of ADULT ADVERSE EXPERIENCES
August, 1992

ABBREVIATIONS

Abbreviations utilized in the table:

ULN = Upper Limit of Normal	LLN = Lower Limit of Normal
Rx = Therapy	Req = Required
Mod = Moderate	IV = Intravenous
ADL = Activities of Daily Living	Dec = Decreased

ESTIMATING SEVERITY GRADE

For abnormalities NOT found elsewhere on the Tox Table, use the scale below to estimate grade of severity:

GRADE 1 Mild	Transient or mild discomfort; no limitation in activity; no medical intervention/therapy required
GRADE 2 Moderate	Mild to moderate limitation in activity - some assistance may be needed; no or minimal medical intervention/therapy required
GRADE 3 Severe	Marked limitation in activity, some assistance usually required; medical intervention/therapy required, hospitalizations possible
GRADE 4 Life-threatening	Extreme limitation in activity, significant assistance required; significant medical intervention/therapy required, hospitalization or hospice care probable

SERIOUS OR LIFE-THREATENING AEs

ANY clinical event deemed by the clinician to be serious or life-threatening should be considered a grade 4 adverse experience. Clinical events considered to be serious or life-threatening include, but are not limited to:

seizures, coma, tetany, diabetic ketoacidosis, disseminated intravascular coagulation,

diffuse petechiae, paralysis, acute psychosis

MISCELLANEOUS

- When two values are used to define the criteria for each parameter, the lowest values will be first.
- Parameters are generally grouped by body system.
- Some protocols may have additional protocol specific grading criteria.

PARAMETER	GRADE 1<br>MILD	GRADE 2<br>MODERATE	GRADE 3<br>SEVERE	GRADE 4<br>POTENTIALLY LIFE-THREATENING
HEMATOLOGY				
Hemoglobin	8.0 g/dL - 9.4 g/dL	7.0 g/dL - 7.9 g/dL	6.5 g/dL - 6.9 g/dL	<6.5 g/dL
Absolute Neutrophil Count	1000 - 1500/mm^3	750 - 999/mm^3	500 - 749/mm^3	<500/mm^3
Platelets	75,000 - 99,000/mm^3	50,000 - 74,999/mm^3	20,000 - 49,999/mm^3	<20,000/mm^3
Prothrombin Time (PT)	>1.0 - 1.25 x ULN	>1.25 - 1.5 x ULN	>1.5 - 3.0 x ULN	>3 x ULN
PTT	>1.0 - 1.66 x ULN	>1.66 - 2.33 x ULN	>2.33 - 3.0 x ULN	>3.0 x ULN
Methemoglobin	5.0 - 10.0%	10.1 - 15.0%	15.1 - 20.0%	>20%
CHEMISTRIES				
SODIUM				
Hyponatremia	130 - 135 meq/L	123 - 129 meq/L	116 - 122 meq/L	<116 meq/L
Hypernatremia	146 - 150 meq/L	151 - 157 meq/L	158 – 165 meq/L	>165 meq/L
POTASSIUM				
Hypokalemia	3.0 - 3.4 meq/L	2.5 - 2.9 meq/L	2.0 - 2.4 meq/L	<2.0 meq/L
Hyperkalemia	5.6 - 6.0 meq/L	6.1 - 6.5 meq/L	6.6 - 7.0 meq/L	>7.0 meq/L
PHOSPHATE				

PARAMETER	GRADE 1<br>MILD	GRADE 2<br>MODERATE	GRADE 3<br>SEVERE	GRADE 4<br>POTENTIALLY LIFE-THREATENING
Hypophosphatemia	2.0 - 2.4 mg/dL	1.5 - 1.9 mg/dL	1.0 - 1.4 mg/dL	<1.0 mg/dL
CALCIUM (corrected for albumin)				
Hypocalcemia	7.8 - 8.4 mg/dL	7.0 - 7.7 mg/dL	6.1 - 6.9 mg/dL	<6.1 mg/dL
Hypercalcemia	10.6 - 11.5 mg/dL	11.6 - 12.5 mg/dL	12.6 - 13.5 mg/dL	>13.5 mg/dL
MAGNESIUM				
Hypomagnesemia	1.2 - 1.4 meq/L	0.9 - 1.1 meq/L	0.6 - 0.8 meq/L	<0.6 meq/L
BILIRUBIN				
Hyperbilirubinemia	>1.0 - 1.5 x ULN	>1.5 - 2.5 x ULN	>2.5 - 5 x ULN	>5 x ULN
GLUCOSE				
Hypoglycemia	55 - 64 mg/dL	40 - 54 mg/dL	30 - 39 mg/dL	<30 mg/dL
Hyperglycemia (nonfasting and no prior diabetes)	116 - 160 mg/dL	161 - 250 mg/dL	251 - 500 mg/dL	>500 mg/dL
Triglycerides	————	400 - 750 mg/dL	751 - 1200 mg/dL	>1200 mg/dL
Creatinine	>1.0 - 1.5 x ULN	>1.5 - 3.0 x ULN	>3.0 - 6.0 x ULN	>6.0 x ULN
URIC ACID				
Hyperuricemia	7.5 - 10.0 mg/dL	10.1 - 12.0 mg/dL	12.1 – 15.0 mg/dL	>15.0 mg/dL

PARAMETER	GRADE 1<br>MILD	GRADE 2<br>MODERATE	GRADE 3<br>SEVERE	GRADE 4<br>POTENTIALLY LIFE-THREATENING
LIVER TRANSAMINASE (LFTs)				
AST (SGOT)	1.25 - 2.5 x ULN	>2.5 - 5.0 x ULN	>5.0 - 10.0 x ULN	>10.0 x ULN
ALT (SGPT)	1.25 - 2.5 x ULN	>2.5 - 5.0 x ULN	>5.0 - 10.0 x ULN	>10.0 x ULN
GGT	1.25 - 2.5 x ULN	>2.5 - 5.0 x ULN	>5.0 - 10.0 x ULN	>10.0 x ULN
Alk Phos	1.25 - 2.5 x ULN	>2.5 - 5.0 x ULN	>5.0 - 10.0 x ULN	>10.0 x ULN
PANCREATIC ENZYMES				
Amylase	>1.0 - 1.5 x ULN	>1.5 - 2.0 x ULN	>2.0 - 5.0 x ULN	>5.0 x ULN
Pancreatic amylase	>1.0 - 1.5 x ULN	>1.5 - 2.0 x ULN	>2.0 - 5.0 x ULN	>5.0 x ULN
Lipase	>1.0 - 1.5 x ULN	>1.5 - 2.0 x ULN	>2.0 - 5.0 x ULN	>5.0 x ULN
CARDIOVASCULAR				
Cardiac Arrhythmia	————	Asymptomatic; transient dysrhythmia; no Rx req	Recurrent/persistent dysrhythmia; symptomatic; Rx req	Unstable dysrhythmia, hospitalization and Rx req
Hypotension	Transient orthostatic hypotension, no Rx	Symptoms correctable with oral fluid Rx	IV fluid req, no hospitalization req	Hospitalization req

PARAMETER	GRADE 1<br>MILD	GRADE 2<br>MODERATE	GRADE 3<br>SEVERE	GRADE 4<br>POTENTIALLY LIFE-THREATENING
Hypertension	Transient, increase >20 mmHg; no Rx	Recurrent; chronic increase >20 mmHg, Rx req	Acute Rx req; outpatient hospitalization possible	Hospitalization req
Pericarditis	Minimal effusion	Mild/mod asymptomatic effusion, no Rx	Symptomatic effusion, pain, EKG changes	Tamponade OR pericardiocentesis OR surgery req
Hemorrhage, Blood Loss	————	Mildly symptomatic, no Rx required	Gross blood loss OR 1-2 units transfused	Massive blood loss OR >2 units transfused
GASTROINTESTINAL				
Nausea	Mild OR transient; reasonable intake maintained	Mod discomfort OR intake decreased for <3 days	Severe discomfort OR minimal intake for ≥3 days	Hospitalization req
Vomiting	Mild OR transient; 2-3 episodes per day OR mild vomiting lasting <1 week	Mod OR persistent; 4-5 episodes per day; OR vomiting lasting ≥1 week	Severe vomiting of all food/fluids in 24 hrs OR orthostatic hypotension OR IV Rx req	Hypotensive shock OR hospitalization req for IV req
Diarrhea	Mild OR transient; 3-4 loose stools per day OR mild diarrhea lasting <1 week	Mod OR persistent; 5-7 loose stools per day OR diarrhea lasting ≥1 week	Bloody diarrhea; OR orthostatic hypotension OR >7 loose stools/day OR IV Rx req	Hypotensive shock OR hospitalization req

PARAMETER	GRADE 1<br>MILD	GRADE 2<br>MODERATE	GRADE 3<br>SEVERE	GRADE 4<br>POTENTIALLY LIFE-THREATENING
Oral Discomfort/Dysphagia	Mild discomfort, no difficulty swallowing	Difficulty swallowing but able to eat and drink	Unable to swallow solids	Unable to drink fluids; IV fluids req
Constipation	Mild	Moderate	Severe	Distention with vomiting
RESPIRATORY				
Cough (for aerosol studies)	Transient; no Rx	Treatment associated cough; inhaled bronchodilator	Uncontrolled cough; systemic Rx req	————
Bronchospasm Acute	Transient; no Rx; FEV1 70% - 80% (or peak flow)	Rx req; normalizes with bronchodilator; FEV1 50% - 70% (or peak flow)	No normalization with bronchodilator; FEV1 25% - 50% (or peak flow), retractions	Cyanosis; FEV1 <25% (or peak flow) OR intubated
Dyspnea	Dyspnea on exertion	Dyspnea with normal activity	Dyspnea at rest	Dyspnea requiring O_2
NEUROLOGIC				
Neuro-cerebellar	Slight incoordination OR dysdiadochokinesia	Intention tremor OR dysmetria OR slurred speech OR nystagmus	Ataxia requiring assistance to walk or arm incoordination interfering with ADLs	Unable to stand

PARAMETER	GRADE 1<br><br>MILD	GRADE 2<br><br>MODERATE	GRADE 3<br><br>SEVERE	GRADE 4<br><br>POTENTIALLY LIFE-THREATENING
Neuro-psych/mood	————	————	Severe mood changes requiring medical intervention	Acute psychosis req hospitalization
Paresthesia (burning, tingling, etc.)	Mild discomfort; no Rx req	Mod discomfort; non-narcotic analgesia req	Severe discomfort; OR narcotic analgesia req with symptomatic improvement	Incapacitating; OR not responsive to narcotic analgesia
Neuro-motor	Mild weakness in muscle of feet but able to walk and/or mild increase or decrease in reflexes	Mod weakness in feet (unable to walk on heels and/or toes), mild weakness in hands, still able to do most hand tasks and/or loss of previously present reflex or development of hyperreflexia and/or unable to do deep knee bends due to weakness	Marked distal weakness (unable to dorsiflex toes or foot drop), and mod proximal weakness, e.g., in hands interfering with ADLs and/or requiring assistance to walk and/or unable to rise from chair unassisted	Confined to bed or wheel chair because of muscle weakness

PARAMETER	GRADE 1<br><br>MILD	GRADE 2<br><br>MODERATE	GRADE 3<br><br>SEVERE	GRADE 4<br><br>POTENTIALLY LIFE-THREATENING
Neuro-sensory	Mild impairment (dec sensation, e.g., vibratory, pinprick, hot/cold in great toes) in focal area or symmetrical distribution	Mod impairment (mod dec sensation, e.g., vibratory, pinprick, hot/cold to ankles) and/or joint position or mild impairment that is not symmetrical	Severe impairment (dec or loss of sensation to knees or wrists) or loss of sensation of at least mod degree in multiple different body areas (i.e., upper and lower extremities)	Sensory loss involves limbs and trunk
URINALYSIS				
Proteinuria				
Spot urine	1+	2 – 3+	4+	Nephrotic syndrome
24-hour urine	200 mg – 1 g loss/day OR <0.3% OR <3 g/l	>1 – 2 g loss/day OR 0.3 – 1.0% OR 3 – 10 g/l	>2 – 3.5 g loss/day OR >1.0% OR >10 g/l	Nephrotic syndrome OR >3.5 g loss/day
Gross Hematuria	Microscopic only	Gross, no clots	Gross plus clots	Obstructive OR transfusion req
MISCELLANEOUS				
Fever	37.7 – 38.5C OR 100.0 – 101.5F	38.6 – 39.5C OR 101.6 – 102.9F	39.6 – 40.5C OR 103 – 105F	>40.5C OR oral >12 hours >105F

PARAMETER	GRADE 1<br>MILD	GRADE 2<br>MODERATE	GRADE 3<br>SEVERE	GRADE 4<br>POTENTIALLY LIFE-THREATENING
Headache	Mild; no Rx req	Mod; or non-narcotic analgesia Rx	Severe; OR responds to initial narcotic Rx	Intractable; OR requiring repeated narcotic Rx
Allergic Reaction	Pruritus without rash	Localized urticaria	Generalized urticaria angiodema	Anaphylaxis
Cutaneous/Rash/Dermatitis	Erythema, pruritus	Diffuse maculopapular rash OR dry desquamation	Vesiculation OR moist desquamation OR ulceration	ANY ONE: mucous membrane involvement, suspected Stevens-Johnson (TEN), erythema multiforme, necrosis req surgery, exfoliative dermatitis
Local Reaction	Erythema	Induration <10 mm OR inflammation OR phlebitis	Induration >10 mm OR ulceration	Necrosis of skin (2° parenteral Rx – not vaccination or skin test)
Fatigue	Normal activity reduced 25%	Normal activity reduced 25-50%	Normal activity reduced >50%; cannot work	Unable to care for self

APPENDIX V: LC002-DERMAVIR FORMULATION RECORD

DermaVir (LC002) Formulation Record
MANUFACTURING CERTIFICATE

The following is to be filled out for each formulation of DermaVir. Refer to clinical protocol for the appropriate study-specific formulation volumes and dose groups.

Date of formulation: _______________

Pharmacist: _______________________ Clinical Site: ________________________

Patient-ID: _______________________ Study cohort and dose (mL): ______/________

1. Record the lot numbers of the respective DermaVir components:

**Component**	**Manufacturer's Lot #**
Plasmid DNA	
PEIm	
Dextrose	

2. Formulation:
Note: A single formulation is required for subjects from Cohorts 1 and 2; for Cohort 3, two separate formulations (A and B) are required to manufacture the total volume of LC002. Formulations A and B represent separate "batches" of product with different batch #.

Procedures	Formulation A	Formulation B (Cohort 3 subjects only)
Record volume of dextrose added to DNA vial		
Record volume of dextrose added to PEIm vial		
Record volume of PEIm+dextrose added to DNA+dextrose vial		
Record batch # (MMDDYY-XX*)		

*XX = sequence of formulation for that day, e.g. 1st formulated dose completed = 01, etc.

3. Record time of formulation (24h clock) here _____:____h.
4. Label all final vials with appropriate use-by date and time (3 hours later), batch number and patient ID and deliver to the clinic.

Prepared by: _______________________________ Date: ____________________

APPENDIX VI: DECLARATION OF HELSINKI

**The ``Declaration of Helsinki" states as follows:
Recommendations Guiding Physicians in Biomedical Research Involving Human Subjects**

Introduction

It is the mission of the physician to safeguard the health of the people. His or her knowledge and conscience are dedicated to the fulfillment of this mission.

The Declaration of Geneva of the World Medical Association binds the physician with the words, ``The health of my patient will be my first consideration," and the International Code of Medical Ethics declares that, ``A physician shall act only in the patient's interest when providing medical care which might have the effect of weakening the physical and mental condition of the patient."

The purpose of biomedical research involving human subjects must be to improve diagnostic, therapeutic and prophylactic procedures and the understanding of the aetiology and pathogenesis of disease.

In current medical practice most diagnostic, therapeutic or prophylactic procedures involve hazards. This applies especially to biomedical research. Medical progress is based on research which ultimately must rest in part on experimentation involving human subjects.

In the field of biomedical research a fundamental distinction must be recognized between medical research in which the aim is essentially diagnostic or therapeutic for a patient, and medical research, the essential object of which is purely scientific and without implying direct diagnostic or therapeutic value to the person subjected to the research.

Special caution must be exercised in the conduct of research which may affect the environment, and the welfare of animals used for research must be respected.
Because it is essential that the results of laboratory experiments be applied to human beings to further scientific knowledge and to help suffering humanity, the World Medical Association has prepared the following recommendations as a guide to every physician in biomedical research involving human subjects. They should be kept under review in the future. It must be stressed that the standards as drafted are only a guide to physicians all over the world. Physicians are not relieved from criminal, civil and ethical responsibilities under the laws of their own countries.

I. Basic Principles

1. Biomedical research involving human subjects must conform to generally accepted scientific principles and should be based on adequately performed laboratory and animal experimentation and on a thorough knowledge of the scientific literature.

2. The design and performance of each experimental procedure involving human subjects should be clearly formulated in an experimental protocol which should be transmitted for consideration, comment and guidance to a specially appointed committee independent of the investigator and the sponsor provided that this independent committee is in conformity with the laws and regulations of the country in which the research experiment is performed.

3. Biomedical research involving human subjects should be conducted only by scientifically qualified persons and under the supervision of a clinically competent medical person. The responsibility for the human subject must always rest with a medically qualified person and never rest on the subject of the research, even though the subject has given his or her consent.

4. Biomedical research involving human subjects cannot legitimately be carried out unless the importance of the objective is in proportion to the inherent risk to the subject.

5 Every biomedical research project involving human subjects should be preceded by careful assessment of predictable risks in comparison with foreseeable benefits to the subject or to others. Concern for the interests of the subject must always prevail over the interests of science and society.

6. The right of the research subject to safeguard his or her integrity must always be respected. Every precaution should be taken to respect the privacy of the subject and to minimize the impact of the study on the subject's physical and mental integrity and on the personality of the subject.

7. Physicians should abstain from engaging in research projects involving human subjects unless they are satisfied that the hazards involved are believed to be predictable. Physicians should cease any investigation if the hazards are found to outweigh the potential benefits.

8. In publication of the results of his or her research, the physician is obliged to preserve the accuracy of the results. Reports of experimentation not in accordance with the principles laid down in this Declaration should not be accepted for publication.

9. In any research on human beings, each potential subject must be adequately informed of the aims, methods, anticipated benefits and potential hazards of the study and the discomfort it may entail. He or she should be informed that he or she is at liberty to abstain from participation in the study and that he or she is free to withdraw his or her consent to participation at any time. The physician should then obtain the subject's freely-given informed consent, preferably in writing.

10. When obtaining informed consent for the research project the physician should be particularly cautious if the subject is in a dependent relationship to him or her or may consent under duress. In that case the informed consent should be obtained by a physician who is not engaged in the investigation and who is completely independent of this official relationship.

11. In case of legal incompetence, informed consent should be obtained from the legal guardian in accordance with national legislation. Where physical or mental incapacity makes it impossible to obtain informed consent, or when the subject is a minor, permission from the responsible relative replaces that of the subject in accordance with national legislation.

12. Whenever the minor child is in fact able to give a consent, the minor's consent must be obtained in addition to the consent of the minor's legal guardian.

    The research protocol should always contain a statement of the ethical considerations involved and should indicate that the principles enunciated in the present Declaration are complied with.

II. Medical Research Combined with Professional Care (Clinical Research)

1. In the treatment of the sick person, the physician must be free to use a new diagnostic and therapeutic measure, if in his or her judgment it offers hope of saving life, reestablishing health or alleviating suffering.

2. The potential benefits, hazards and discomfort of a new method

should be weighed against the advantages of the best current diagnostic and therapeutic methods.

3. In any medical study, every patient--including those of a control group, if any--should be assured of the best proven diagnostic and therapeutic method.

4. The refusal of the patient to participate in a study must never interfere with the physician-patient relationship.

5. If the physician considers it essential not to obtain informed consent, the specific reasons for this proposal should be stated in the experimental protocol for transmission to the independent committee (I, 2).

6. The physician can combine medical research with professional care, the objective being the acquisition of new medical knowledge, only to the extent that medical research is justified by its potential diagnostic or therapeutic value for the patient.

III. Non-Therapeutic Biomedical Research Involving Human Subjects (Non-Clinical Biomedical Research)

1. In the purely scientific application of medical research carried out on a human being, it is the duty of the physician to remain the protector of the life and health of that person on whom biomedical research is being carried out.

2. The subjects should be volunteers--either healthy persons or patients for whom the experimental design is not related to the patient's illness.

3. The investigator or the investigating team should discontinue the research if in his/her or their judgment it may, if continued, be harmful to the individual.

4. In research on man, the interest of science and society should never take precedence over considerations related to the well-being of the subject.

(Collection of information requirements approved by the Office of Management and Budget under control number 0910-0014)
[52 FR 8831, Mar. 19, 1987, as amended at 52 FR 23031, June 17, 1987; 56 FR 22113, May 14, 1991]
